# Metal-Adapted Bacteria Isolated From Wastewaters Produce Biofilms by Expressing Proteinaceous Curli Fimbriae and Cellulose Nanofibers

**DOI:** 10.3389/fmicb.2018.01334

**Published:** 2018-06-25

**Authors:** M. K. Mosharaf, M. Z. H. Tanvir, M. M. Haque, M. A. Haque, M. A. A. Khan, A. H. Molla, Mohammad Z. Alam, M. S. Islam, M. R. Talukder

**Affiliations:** ^1^Department of Environmental Science, Faculty of Agriculture, Bangabandhu Sheikh Mujibur Rahman Agricultural University, Gazipur, Bangladesh; ^2^Department of Agro-Processing, Faculty of Agriculture, Bangabandhu Sheikh Mujibur Rahman Agricultural University, Gazipur, Bangladesh; ^3^Department of Plant Pathology, Faculty of Agriculture, Bangabandhu Sheikh Mujibur Rahman Agricultural University, Gazipur, Bangladesh; ^4^Bangladesh Jute Research Institute, Dhaka, Bangladesh

**Keywords:** wastewater, biofilm, extracellular polymeric substance, cellulose, curli fimbriae, heavy metal

## Abstract

Bacterial biofilm plays a pivotal role in bioremediation of heavy metals from wastewaters. In this study, we isolated and identified different biofilm producing bacteria from wastewaters. We also characterized the biofilm matrix [i.e., extracellular polymeric substances (EPS)] produced by different bacteria. Out of 40 isolates from different wastewaters, only 11 (27.5%) isolates (static condition at 28°C) and 9 (22.5%) isolates (agitate and static conditions at 28 and 37°C) produced air–liquid (AL) and solid–air–liquid (SAL) biofilms, respectively, only on salt-optimized broth plus 2% glycerol (SOBG) but not in other media tested. Biomass biofilms and bacteria coupled with AL biofilms were significantly (*P* ≤ 0.001) varied in these isolates. *Escherichia coli* (isolate ENSD101 and ENST501), *Enterobacter asburiae* (ENSD102), *Enterobacter ludwigii* (ENSH201), *Pseudomonas fluorescens* (ENSH202 and ENSG304), uncultured *Vitreoscilla* sp. (ENSG301 and ENSG305), *Acinetobacter lwoffii* (ENSG302), *Klebsiella pneumoniae* (ENSG303), and *Bacillus thuringiensis* (ENSW401) were identified based on 16S rRNA gene sequencing. Scanning electron microscope (SEM) images revealed that biofilm matrix produced by *E. asburiae* ENSD102, uncultured *Vitreoscilla* sp. ENSG301, *A. lwoffii* ENSG302, and *K. pneumoniae* ENSG303 are highly fibrous, compact, and nicely interlinked as compared to the biofilm developed by *E. ludwigii* ENSH201 and *B. thuringiensis* ENSW401. X-ray diffraction (XRD) results indicated that biofilm matrix produced by *E. asburiae* ENSD102, uncultured *Vitreoscilla* sp. ENSG301, and *A. lwoffii* ENSG302 are non-crystalline amorphous nature. Fourier transform infrared (FTIR) spectroscopy showed that proteins and polysaccharides are the main components of the biofilms. Congo red binding results suggested that all these bacteria produced proteinaceous curli fimbriae and cellulose-rich polysaccharide. Production of cellulose was also confirmed by Calcofluor binding- and spectrophotometric assays. *E. asburiae* ENSD102, *Vitreoscilla* sp. ENSG301, and *A. lwoffii* ENSG302 were tested for their abilities to form the biofilms exposure to 0 to 2000 mg/L of copper sulfate (for Cu), zinc sulfate (for Zn), lead nitrate (for Pb), nickel chloride (for Ni), and potassium dichromate (for Cr), several concentrations of these metals activated the biofilm formation. The polysaccharides is known to sequester the heavy metals thus, these bacteria might be applied to remove the heavy metals from wastewater.

## Introduction

Discharge of untreated wastewater into the rivers, cannels, lakes, and ponds is one of the major causes of water pollution. Generally, wastewater contains toxic heavy metals, synthetic dyes, and other hazardous substances (Jin et al., 2007; Das et al., 2011; Islam et al., 2011; Saratale et al., 2011; Sheikh et al., 2017), which pose threat to human health, fish, crops, and overall biodiversity (Islam et al., 2014, 2015; Naser et al., 2014; Ahmed et al., 2016; Alam et al., 2017). All around the world, numerous physico-chemical methods (e.g., chemical precipitation, oxidation, reduction, activated carbon, ion-exchange, reverse osmosis, membrane filtration, and evaporation) are being practiced to treat the wastewater. However, most of these methods are expensive, ineffective, and required high energy and produced large amount of sludge with hazardous by-products (Ahluwalia and Goyal, 2007; Dixit et al., 2015). By contrast, microbial-based techniques are eco-friendly, economic, and effectively detoxify the persistent organic pollutants (POPs), petroleum products, explosives, dyes, and metals from wastewater (Singh et al., 2006; Saratale et al., 2011; Edwards and Kjellerup, 2013; Elekwachi et al., 2014; Dixit et al., 2015; Mitra and Mukhopadhyay, 2016).

Biofilms are structured, surface-adherent, multicellular, microbial communities. Biofilms consist mainly of cells embedded in a self-produced extracellular polymeric substances [EPS (Costerton et al., 1999; Donlan and Costerton, 2002; Flemming and Wingender, 2010)]. EPS comprises polysaccharides, including cellulose nanofibers (Zogaj et al., 2001; Solano et al., 2002; Haque et al., 2009; Römling and Galperin, 2015) and sucrose-derived glucans and fructans (Wingender et al., 2001), proteins, such as lectins, Bap-like proteins (Lasa and Penadé, 2006), and proteinaceous appendages mainly curli fimbriae (Prigent-Combaret et al., 2000; White et al., 2003; Zogaj et al., 2003), extracellular DNA (Whitchurch et al., 2002; Liang et al., 2010), lipids (e.g., surfactin, viscosin, and emulsan) (Conrad et al., 2003), surfactants (e.g., rhamnolipids) (Davey et al., 2003), and other biopolymers, including humic substances (Martín-Cereceda et al., 2001). The specific contents of the EPS controls biofilm morphology and stability (Flemming and Wingender, 2010). However, composition of the EPS vary between biofilms, species, surface on which biofilms are formed and environmental conditions, including availability of the nutrients, temperature, and oxygen tension (Prouty and Gunn, 2003; Haque et al., 2012; Koechler et al., 2015).

Bacterial biofilm matrix, i.e., EPS play significant roles compared with their free-living planktonic counterparts, including protection of the cells from adverse environmental stresses (e.g., high concentration of toxic chemicals, changes in pH, temperature, salt concentration, and water content), ability to communicate through expression of signal molecules, exchange genetic materials, and persistence in different metabolic states (Teitzel and Parsek, 2003; Hall-Stoodley et al., 2004; Kaplan, 2010; McDougald et al., 2012; Koechler et al., 2015; Mitra and Mukhopadhyay, 2016; Haque et al., 2017). Among the contents of the EPS, specifically the polysaccharide binds to heavy metals (Ferris et al., 1989; Teitzel and Parsek, 2003; van Hullebusch et al., 2003; Li and Yu, 2014). Despite these advantages, bacterial biofilms have been appreciated and applied to remove xenobiotic compounds (Seo et al., 2009; Payne et al., 2011; Edwards and Kjellerup, 2013) and heavy metal ions (Huang et al., 2000; Labrenz et al., 2000; Chang et al., 2006; Muñoz et al., 2006; Singh et al., 2006; Pal and Paul, 2008; Yamaga et al., 2010; Das et al., 2012; Fida et al., 2012). Cellulose nanofibers have been shown to use as a scaffold for tissue engineering (Klemm et al., 2001; Maneerung et al., 2007).

Only a few bacterial biofilms, including *Acinetobacter calcoaceticus*, *Bacillus subtilis*, *B. cereus*, *Escherichia coli*, *Pseudomonas putida*, *P. aeruginosa*, and *Rhodococcus* sp. have been found to remove the toxic heavy metals (Wagner-Döbler et al., 2000; Al-Awadhi et al., 2003; Pal and Paul, 2008; Cristina et al., 2009; Fang et al., 2011; Sundar et al., 2011). More diverse biofilms is more efficient for bioremediation of heavy metals (von Canstein et al., 2002; Edwards and Kjellerup, 2013). The objective of this study was to isolate and identify the biofilm producing bacteria from dyeing, composite (mixture of household and different industries), garments, washing plant, and tannery wastewater of Bangladesh. In this study, we also characterized the matrix of the biofilms (i.e., EPS) produced by different bacteria by means of scanning electron microscope (SEM), Fourier transform infrared (FTIR) spectroscopy, X-ray diffraction (XRD), different binding (e.g., Congo red and Calcufluor binding assays) and spectrophotometric assays. The effect of bacterial biofilms in resistance to toxic heavy metals is well documented (Teitzel and Parsek, 2003; Harrison et al., 2005). However, information regarding the role of toxic heavy metals on biofilm formation is only poorly understood (Koechler et al., 2015). Therefore, it is aims to quantify the effect of different concentrations (0, 500, 750, 1000, 1250, 1500, 1750, and 2000 mg/L) of copper sulfate (for Cu), zinc sulfate (for Zn), lead nitrate (for Pb), nickel chloride (for Ni), and potassium dichromate (for Cr) on bacterial growth in agitate condition and biofilm formation in static condition in some selected bacteria. This study will contribute toward understanding the potential of different bacterial biofilms in bioremediation of toxic heavy metals presence in contaminated wastewaters.

## Materials and Methods

### Sampling and Physico-Chemical Properties of the Wastewaters

Dyeing, composite (household plus different industrial wastewaters), garments, washing plant’s wastewaters were collected from Gazipur city areas of Bangladesh, while tannery wastewater was collected from Hazaribagh of Dhaka city, Bangladesh. The samples were collected in cleaned and sterilized screw cap bottles, and cold chain was maintained during transportation to the laboratory. Collected samples were stored at 4°C before analysis. Color and odor of the samples were noted. Total dissolve solid (TDS), salinity, and electrical conductivity (EC) were measured by Conductivity meter (Model: DDSJ-308A). Water pH was determined by the digital pH meter (model: HI 8424, HANNA). Water temperature and dissolved oxygen (DO) were measured during sample collection with the help of digital thermometer and digital DO meter (Model: HI 8424, HANNA), respectively. Copper (Cu), zinc (Zn), lead (Pb), chromium (Cr), and nickel (Ni) in different wastewater samples were determined by atomic absorption spectrophotometer (Model- AA-7000, Shimadzu, Japan) followed by procedures of American Public Health Association [APHA] (1998). Physico-chemical characteristics of various wastewaters are presented in Supplementary Table S1.

### Isolation and Purification of Bacteria

Each wastewater sample was serially diluted with sterile distilled water then streaked on yeast extract peptone (YP) (1% of peptone, 0.5% of yeast extract, pH 6.8) agar (1.5%) plates. The plates were incubated at 28°C. After 24 h incubation, morphologically distinct (e.g., color, size, and shape) eight colonies from each sample were transferred to the fresh YP agar plate by the sterile toothpicks. Pure culture of each isolate was made by repeated streaking method and used for further studies.

### Screening of Biofilm Producing Isolates

Initially a single colony of each isolate was grown in YP broth at 28°C in shaking condition (180 rpm) overnight and diluted [1:100 (ca. 10^6^ colony forming unit (CFU)/mL)]. Then 50 μL diluted culture were inoculated in glass test tubes (Pyrex, flat bottom, Glassco, United Kingdom) containing 5 mL of salt-optimized broth (SOB) plus glycerol (SOBG) broth (per liter, 20 g of tryptone, 5 g of yeast extract, 0.5 g of NaCl, 2.4 g of MgSO_4_.7H_2_O, 0.186 g of KCl, and 50 ml of 40% glycerol), YP, Luria Bertani (LB), King’s B (KB), yeast peptone dextrose adenine (YPDA), and M63 glycerol minimal medium (per liter, 2.5 g of NaCl, 3 g of KH_2_PO_4_, 7 g of K_2_HPO_4_, 2 g of (NH_4_)_2_SO_4_, 0.5 mg of FeSO_4_, 2 g of thiamine hydrochloride, and 2 g of glycerol). Then each test tube was incubated at two different temperatures (28 or 37°C) in static or agitate (150 rpm) condition. After 72 h incubation, solid–air–liquid (SAL) and air–liquid (AL) biofilm producing isolates were identified visually.

### Quantification of AL Biofilms

Biomass of the rigid AL biofilms was extracted from the liquid medium and quantified as described in Haque et al. (2012) with a few modifications. In brief, each biofilm was gently removed from the glass test tube and washed two to three times with sterile distilled water. Then 1.5 mL sterile distilled water and 20 glass beads (3 mm) were added to each glass test tube. Each biofilm was detached by vortexing (50 s) at the highest speed. Then optical density (OD) was measured by reading the absorbance at 600 nm with an UV spectrophotometer (Ultrospec 3000, Pharmacia Biotech, Cambridge, United Kingdom). Biomass of the fragile AL biofilms was extracted/detached from the liquid and quantified as follows: after 72 h of incubation in static condition, 1 mL of planktonic culture, i.e., the culture beneath the AL biofilms was carefully collected by inserting the pipette tips, and OD (OD_600_) was determined by the UV spectrophotometer. Then each fragile AL biofilms was vigorously vortexed with 1 mL planktonic culture and 20 glass beads (3 mm), and OD_600_ was measured. Afterward, the OD_600_ of planktonic culture was subtracted from OD_600_ of biomass of fragile AL biofilm plus planktonic culture. This would provide the amount of fragile biomass present in the AL biofilm.

### Quantification of SAL Biofilms

After 72 h of incubation at 180 rpm at 28°C, 5.5 mL of 0.05% (w/v) crystal violet (CV) solution was added to each glass test tube then incubated for 45 min. Each test tube was rinsed with three times with sterile distilled water, and CV was eluted using 95% ethanol. The SAL biofilm was quantified by measuring the absorbance at 570 nm using UV spectrophotometer (Ultrospec 3000, Pharmacia, Biotech, Cambridge, United Kingdom).

### Enumeration of Cells Coupled With Biofilms

The rigid AL biofilms were carefully transferred from the broth, washed with sterile distilled water (twice), and then placed in sterile glass test tubes containing 3 mL of YP broth and 40 glass beads. The biofilms were disrupted for 1 min by vortexing, serially diluted, spread on YP agar plates, and incubated at 28°C. After 32 h, the cells were enumerated. The bacterial cells coupled with fragile AL biofilms were counted as follows: first, 1 mL of planktonic culture was gently removed, diluted, then spread on YP agar plates. Second, the fragile AL biofilms were mixed with 3 mL of planktonic culture by vortexing (without glass beads), diluted, and spread on YP agar plates. After 32 h incubation at 28°C, the CFU were counted. Afterward, CFU of the planktonic culture was subtracted from CFU of fragile AL biofilm plus planktonic culture. This would provide the cells present in the fragile AL biofilm.

### Identification of Biofilm Producing Bacterial Strains Using 16S rRNA Gene Sequencing

Extraction of genomic DNAs and gel electrophoresis was done as described in Sambrook et al. (1989). 16S rRNA genes were amplified by polymerase chain reaction (PCR) using the universal bacterial 16S rRNA gene primers 27F (5′-AGAGTTTGATCMTGGCTCAG-3′) and 1492R (5′-GGTTACCTTGTTACGACTT-3′). The PCR amplification conditions were as follows: initial DNA denaturation at 94°C for 5 min followed by 35 cycles of denaturation at 94°C for 30 s, annealing at 57°C for 45 s, and elongation at 72°C for 1.5 min, which was followed by a final extension at 72°C for 10 min. The PCR products were purified with QIAquick^®^ Gel extraction kit (Qiagen), essentially according to the manufacturer’s instructions. Nucleotide sequences were determined from the purified products by using 3500 Genetic Analyzer (Applied Bio-system). Two forward primers 27F (5′-AGAGTTTGATCMTGGCTCAG-3′) and 533F (5′-GTGCCAGCAGCCGCG GTAA-3′) and one reverse primer 1492R (5′-GGTTACCTTGTTACGACTT-3′) were used for the sequencing. The gene sequences of different biofilm-producing bacterial strains were compared using the bioinformatics tool BLASTN (Basic Local Alignment Search for Nucleotide) against the sequences of bacteria available in National Center for Biotechnology Information (NCBI) data banks^1^.

### Construction of Phylogenetic Tree

All sequences were aligned with MUSCLE (Edgar, 2004). Alignments were pruned with G blocks (Castresana, 2000). The evolutionary history was inferred by using the Maximum Likelihood method based on the JTT matrix-based model (Jones et al., 1992). Evolutionary analyses were conducted in MEGA6 (Tamura et al., 2013).

### Scanning Electron Microscopy

A scanning electron microscopy (SEM) (SUI510, Hitachi, Japan) operated at 5.0 KV was used to image biofilm samples after centrifugation (10,000 rpm for 10 min) followed by drying (12 h) at 40°C. Each sample was coated with carbon using a vacuum sputter-coater to improve the conductivity of the sample.

### Acquiring and Analysis of the IR Spectra

The IR spectra of the biofilms were acquired through Perkin Elmer FTIR (Spectrum-2) instrument operated by CPU32M software. Biofilms were removed carefully from SOBG broth after 72 h incubation by pouring the culture into the tube and centrifuged at 13,000 rpm for 20 min. The precipitates after centrifugation were directly used as samples for FTIR scanning; within 450 to 4000 cm^-1^ using triglycine sulfate (TGS) detector. A total of 16 scans at 4 cm^-1^; resolution were accumulated at 0.2 cm/s scanning speed. The supernatant of the SOBG broth was also scanned. The spectrum of the supernatant was subtracted from the sample spectra to present the result. The baseline subtracted biofilms spectra were analyzed by using Perkin Elmer’s proprietary software (Version 10.05.03).

### X-Ray Diffraction Analysis

This study was carried out on a BRUKER D8 X-ray diffractometer with CuKα1 radiation (λ = 1.54056). A continuous scan type diffractograms were recorded between 5.01° and 74.99° (2θ) at a rate of 0.3 s/step with a step size of 0.02° (2θ). A fixed-type anti-scatter slit of 0.10 mm and 1° divergence and receiving slits were used. The measurement temperature was recorded as 25°C.

### Congo Red Binding Assays

Congo red binding assays were done as described by Haque et al. (2017) with a few modifications. In brief, initially each biofilm producing bacteria was grown in YP broth overnight at 28°C in shaking condition (180 rpm). Then 1 mL of culture of each biofilm-producing bacterial strain was collected and centrifuged. The pellet was then diluted 1:100 (ca. 10^5^ CFU/mL). Then 2 μL diluted culture were spotted (five spot in each plate) onto SOBG agar plates containing 40 μg/mL of Congo red (Santa Cruz Biotechnology, United States). The plates were incubated at 28°C in still condition for 48 h, then photographs were taken.

### Calcofluor Binding Assays

Calcofluor binding assays were carried out as described in Haque et al. (2009) with a few modifications. In brief, each biofilm producing bacteria was grown in YP broth overnight at 28°C in agitate condition (180 rpm) and diluted 1:100 (ca. 10^5^ CFU/mL). Then 2 μL of diluted culture of each biofilm-producing bacterial strain were spotted (five spot in each plate) onto SOBG agar plates containing 200 μg/mL of Calcofluor white (Thomas Scientific, Fluka, United States). The plates were incubated at 28°C before being checked under UV light (366 nm). The photographs were taken after 48 h.

### Quantification of Cellulose From Biofilm Producing Bacteria

Cellulose production was quantified from different biofilm producing bacteria as the method described by Haque et al. (2017) with a few modifications. In brief, 2 μL of diluted (overnight grown) culture (ca. 10^5^ CFU/mL) were spotted (15 spots in each plate) onto SOBG Calcoflour agar plates then incubated at 28°C in stationary condition. After 48 h incubation, approximately 3 g of cells from each bacterium were collected in 50-mL polystyrene conical tubes, covered then lyophilized. The lyophilized dry masses were mixed with 5 mL of 8:2:1 acetic acid: nitric acid: distilled water and boiled for 30 min then centrifuged at 15,000 rpm. The cell pellet was transferred to the Corex centrifuged bottles, washed two to three times with sterile distilled water and dried aseptically. The dried pellet was mixed with 200 μL of concentrated H_2_SO_4_ with gentle shaking (50 rpm) for 1.5 h at room temperature. The amount of cellulose was determined (OD_620_) using 800 μL anthrone (Sigma-Aldrich, St. Louis, MO, United States) reagent (0.2 g in 100 mL H_2_SO_4_). The Avicel cellulose (Sigma-Aldrich, St. Louis, MO, United States) was used as standard.

### Heavy Metal Stress on Biofilm Formation

In order to study the effect of heavy metal stress on biofilm formation, 50 μL of cultures were inoculated in 5 mL of magnesium-deprived SOBG broth with different concentrations (0, 500, 750, 1000, 1250, 1500, 1750, and 2000 mg/L) of copper sulfate (CuSO_4_.5H_2_O for Cu), zinc sulfate (ZnSO_4_.7H_2_O for Zn), lead nitrate [Pb(NO_3_)_2_ for Pb], nickel chloride (NiCl_2_ for Ni), and potassium dichromate (K_2_Cr_2_O_7_ for Cr). The test tubes were incubated at 28°C in still condition. After 72 h incubation, the photographs were taken. The biomass biofilms were quantified after 72 h incubation as stated above.

### Statistical Analysis

All the experiments were laid out in a complete randomized design with four replications and repeated at least two times. Analysis of variance and comparison of means were calculated with the statistical package “agricolae” of R software version 3.3.3. The means were compared by using Fisher’s least significance difference (LSD) test (*P* < 0.001).

## Results

### Screening of Biofilm Producing Bacterial Isolates

**FIGURE 1:**
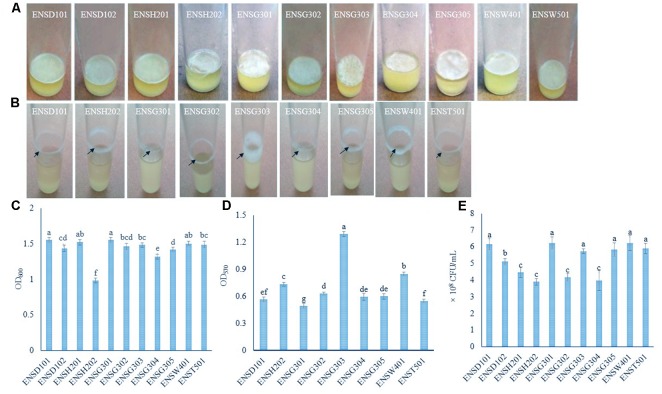
AL **(A)** and SAL **(B)** biofilm formation by different isolates after 72 h incubation at 28°C in static and agitate (150 rpm) condition, respectively. Biomass of AL **(C)** and SAL **(D)** biofilms measured at 600 and 570 nm, respectively. Arrows indicate the SAL biofilm formation. Quantification of bacteria-associated with AL biofilm matrix **(E)**. The values are mean and error bars indicate standard deviation (±SD) of the three independent experiments. Values having different letters are significantly different from each other according to Fisher’s least significant difference (LSD) test (*P* ≤ 0.001).

In order to isolate biofilm producing bacteria from different wastewaters, a total of 40 isolates (8 isolates/sample) were screened. In static condition, 11 (27.5%) isolates, such as ENSD101, ENSD102, ENSH201, ENSH202, ENSG301, ENSG302, ENSG303, ENSG304, ENSG305, ENSW401, and ENST501 were found to produce fragile to rigid AL biofilms at the air–liquid interface (also known as pellicle) in the glass test tubes containing 5 mL of SOBG (**Figure 1A**) after 72 h incubation at 28°C but not in YP, LB, KB, YPDA, and M63 glycerol minimal media (data not shown). AL biofilms developed by ENSD101, ENSD102, ENSH201, ENSG302, ENSG304, ENSW401, and ENST501 had a smooth surface, robust, and cells were not dispersed when the aggregates were agitated. Conversely, AL biofilms produced by ENSH202, ENSG301, ENSG303, and ENSG305 had a rough surface, fragile, and easily dispersed when disturbed. Conversely, at 37°C, all the AL biofilm-forming isolates produced the SAL biofilms on SOBG broth but not in YP, LB, KB, YPDA, and M63 glycerol minimal media after 72 h incubation (data not shown).

In shaking (150 rpm) condition, only nine (22.5%) isolates, including ENSD101, ENSH202, ENSG301, ENSG302, ENSG303, ENSG304, ENSG305, ENSW401, and ENST501 were found to form the SAL biofilms as a ring at the solid–air–liquid interface in the glass test tubes containing 5 mL of SOBG (**Figure 1B**) only but not in YP, LB, KB, YPDA, and M63 glycerol minimal media (data not shown) after 24 h incubation at 28°C. Isolate ENSG303 built a wide and thick SAL biofilm ring than the other isolates. Therefore, SOBG broth was chosen to screen the biofilm producing bacteria from wastewaters. Furthermore, none of the biofilm producing bacterial isolate was found to be impaired in growth rate in SOBG broth and M63 glycerol minimal medium in shaking condition (data not shown).

### Biomass of Biofilms Produced by Different Isolates

AL biomass of biofilm was found to be significantly (*P* ≤ 0.001) differed in these isolates (**Figure 1C**). The isolates of ENSD101 and ENSG301 produced significantly (*P* < 0.001) more AL biomass biofilms (OD_600_ at 1.55) followed by ENSH201 (OD_600_ at 1.52) and ENSW401 (OD_600_ at 1.50). However, the moderate biomass biofilms (OD_600_ at 1.48) was generated by the ENSG303 and ENST501 followed by ENSG302 (OD_600_ at 1.46), ENSD102 (OD_600_ at 1.43), and ENSG305 (OD_600_ at 1.41). Significantly the lowest biomass biofilm (OD_600_ at 0.98) was produced by ENSH202. Like AL biomass biofilm, SAL biomass biofilm was also significantly (*P* ≤ 0.001) differed in these isolates (**Figure 1D**). The isolate ENSG303 produced more SAL biomass biofilm (OD_570_ at 1.29) than the other isolates. The lowest SAL biomass biofilm (OD_570_ at 0.49) was developed by the isolate ENSG301.

### Bacterial Cells Coupled With AL Biofilm Matrix

Bacterial cells coupled with AL biofilm matrix were counted by a serial dilution plating method (**Figure 1E**). The CFU was significantly (*P* ≤ 0.001) higher in the matrix produced by ENSG301 (6.23 × 10^8^) and ENSW401 (6.23 × 10^8^), which were statistically similar with ENSD101 (6.17 × 10^8^), ENSG303 (5.73 × 10^8^), ENSG305 (5.8 × 10^8^), and ENST501 (5.9 × 10^8^). However, the moderate CFU was recorded in the matrix produced by ENSD102. The lowest CFU was detected in the matrix created by ENSH202 (3.90 × 10^8^), which was statistically similar with ENSH201 (4.47 × 10^8^), ENSG302 (4.17 × 10^8^), and ENSG304 (3.97 × 10^8^).

### Identification of Biofilm Producing Bacteria

The 16S rRNA gene from biofilm producing isolates was sequenced, aligned, and the closest match was detected using BLASTN (**Table 1**). However, the isolates of ENSD101 and ENST501 were 99% homologous to *E. coli* (KJ803895.1) with maximum score (score of single best aligned sequence) 2545, ENSG301 and ENSG305 were 98% homologous to uncultured *Vitreoscilla* sp. (LN870312.1) with maximum score 2567, ENSH202 and ENSG304 were 99% homologous to *Pseudomonas fluorescens* (KP126776.1) with maximum score 2615, the isolates of ENSD102, ENSH201, ENSG302, and ENSG303 were 99% homologous to *Enterobacter asburiae* (CP014993.1), *Enterobacter ludwigii* (KM077046.1), *Acinetobacter lwoffii* (KF993657.1), and *Klebsiella pneumoniae* (KF192506.1) with maximum score 2654, 2573, 2468, and 2675, respectively. Conversely the isolate of ENSW401 was 100% homologous to *Bacillus thuringiensis* (JX283457.1) with maximum score 2601. The 16S rRNA gene sequence data were submitted to the NCBI GenBank, and the assigned accession number for uncultured *Vitreoscilla* sp. ENSG301, *A. lwoffii* ENSG302, *E. ludwigii* ENSH201, *B. thuringiensis* ENSW401, *E. coli* ENSD101, *E. asburiae* ENSD102, *K. pneumoniae* ENSG303, and *P. fluorescens* ENSG304 were KU254758, KU254759, KU254760, KU254761, KU254762, KU254763, KU254764, and KU254765, respectively.

**Table 1:** Identification of biofilm forming bacteria.

Strains	Source	Top hit against colony isolate	Accession no.	Maximum score	Maximum identity (%)
ENSD101	Dyeing industry	*Escherichia coli*	KJ803895.1	2545	99
ENSD102		*Enterobacter asburiae*	CP014993.1	2654	99
ENSH201	Composite (household plus different industrial wastewaters)	*Enterobacter ludwigii*	KM077046.1	2573	99
ENSH202		*Pseudomonas fluorescens*	KP126776.1	2615	99
ENSG301	Garments industry	Uncultured *Vitreoscilla* sp.	LN870312.1	2567	98
ENSG302		*Acinetobacter lwoffii*	KF993657.1	2468	99
ENSG303		*Klebsiella pneumoniae*	KF192506.1	2675	99
ENSG304		*P. fluorescens*	KP126776.1	2615	99
ENSG305		Uncultured *Vitreoscilla* sp.	LN870312.1	2567	98
ENSW401	Washing plant industry	*Bacillus thuringiensis*	JX283457.1	2601	100
ENST501	Tannery industry	*E. coli*	KJ803895.1	2545	99


### Phylogenetic Tree

Phylogenetic tree revealed that there were at least seven major clades where each species belonged to a clade representing its genus with species (Supplementary Figure S1). ENSD101 and ENST501 belonged to the same clade as *E. coli*, ENSG301 and ENSG305 formed the same clade as uncultured *Vitreoscilla* sp., ENSH202 and ENSG304 into the same clade as *P. fluorescens*, and ENSD102 and ENSH201 formed another clade as *E. asbuiae* and *E. ludwigii*. However, rest of the isolates, ENSG302, ENSG303, and ENSW401 formed individual clade as *A. lwoffii*, *K. pneumoniae*, and *B. thuringiensis*, respectively.

### Scanning Electron Microscopy

**FIGURE 2:**
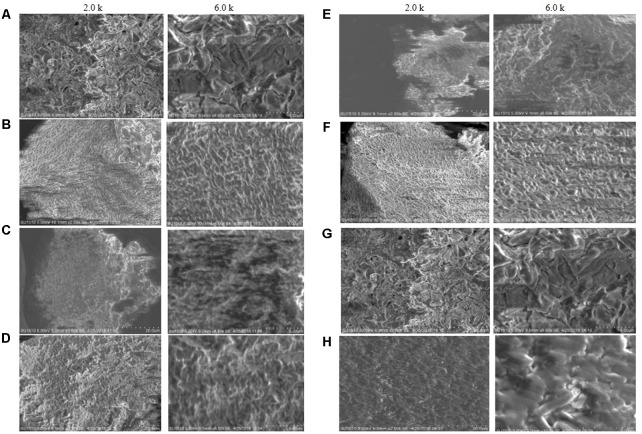
SEM images of the matrix produced by **(A)** *E. coli* ENSD101, **(B)** *E. asburiae* ENSD102, **(C)** *E. ludwigii* ENSH201, **(D)** uncultured *Vitreoscilla* sp. ENSG301, **(E)** *A. lwoffii* ENSG302, **(F)** *K. pneumoniae* ENSG303, **(G)** *P. fluorescens* ENSG304, and **(H)** *B. thuringiensis* ENSW401 with 2.0 and 6.0 k magnifications.

Scanning electron microscopy images of the biofilm matrix are shown in **Figure 2**. Biofilm matrix produced by *E. asburiae* ENSD101 (**Figure 2A**), uncultured *Vitreoscilla* sp. ENSG301 (**Figure 2B**), *A. lwoffii* ENSG302, and *K. pneumoniae* ENSG303 were highly fibrous, compact, and nicely interlinked as compared to the biofilm developed by *E. coli* ENSD101 and *B. thuringiensis* ENSW401 in resolution of 2.0 k. The images were clearer in high resolution of 6.5 k. Cracks were easily visible in the biofilm matrix generated by *E. ludwigii* ENSH201 and *P. fluorescens* ENSG304 leading to form an indented surface morphology. It may be due to the effect of drying and/or centrifugation. In this study, we were unable to measuring the size of interwoven mesh of microfibrils.

### Fourier Transform Infrared Spectroscopy and X-Ray Diffraction Analysis

**FIGURE 3:**
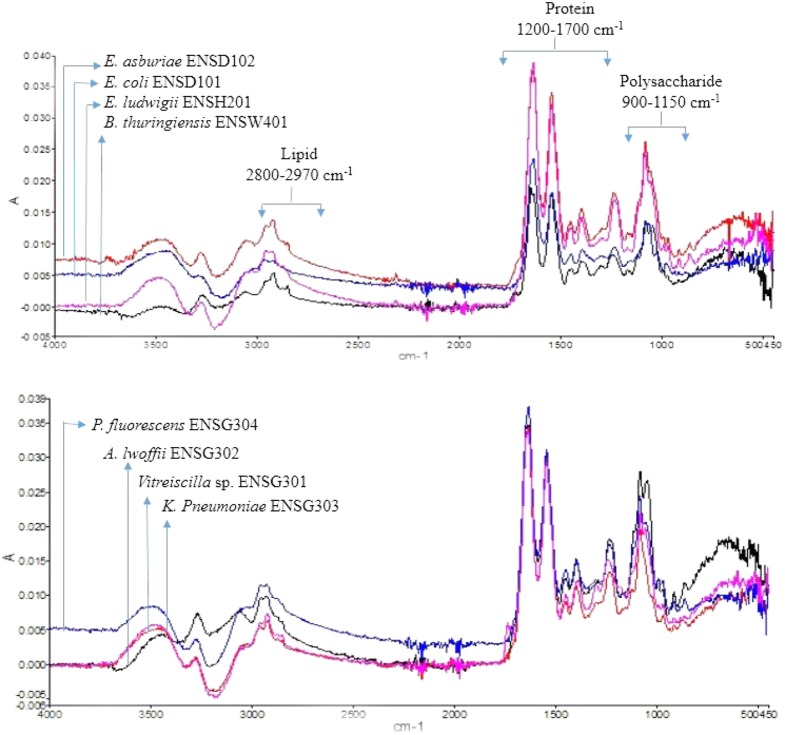
FTIR spectra of the biofilm matrix produced by different bacteria.

**FIGURE 4:**
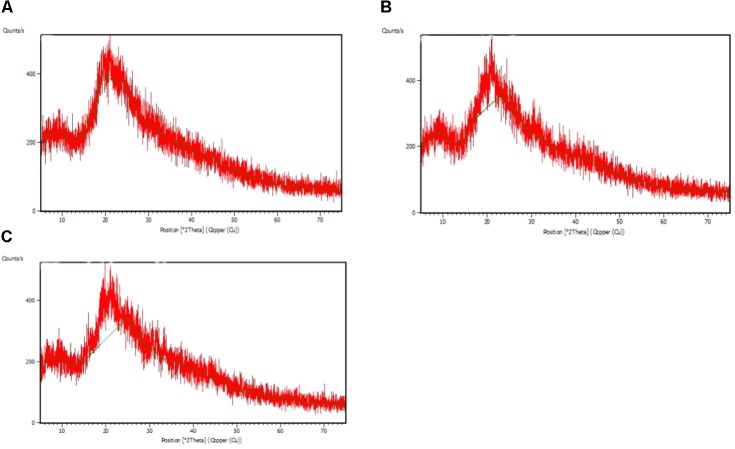
X ray diffraction (XRD) patterns of the matrix of the biofilms produced by *E. asburiae* ENSD102 **(A)**, uncultured *Vitreoscilla* sp. ENSG301 **(B)**, and *A. lwoffii* ENSG302 **(C)**.

The FTIR spectra of the EPS of different biofilms are presented in **Figure 3**. It was observed that all the bacterial EPS were dominant with protein contents producing peaks at amide I (1600–1700 cm^-1^), amide II (1500–1600 cm^-1^), and amide III (1200–1350 cm^-1^) regions. The EPS were also consisted of high content of polysaccharide which produced intense peaks near 900–1150 cm^-1^. The 2800–2970 cm^-1^ domain indicates the presence of small amount of lipids in the EPS. The XRD analyses of the dried biofilm masses were carried out to assess the crystalline/amorphous nature. The XRD of *E. asburiae* ENSD102, uncultured *Vitreoscilla* sp. ENSG301, and *A. lwoffii* ENSG302 biofilms are presented in **Figure 4**. All the XRD patterns exhibit the non-crystalline amorphous nature with producing an extremely broad peak near at around 15–25° (2θ).

### Detection of Curli Fimbriae and Cellulose Nanofibers by Congo Red Binding Assays

**FIGURE 5:**
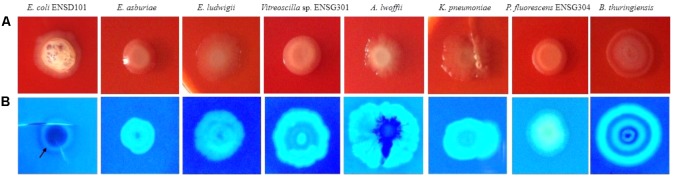
Congo red binding (SOBG agar plates containing 40 μg/mL of Congo red) abilities of the bacteria after 48 h incubation at 28°C in static condition **(A)**. Calcofluor binding capacities of the bacteria (SOBG agar plates containing 200 μg/mL of Calcofluor white) after 48 h incubation at 28°C in static condition **(B)**. Photographs represent one of three experiments, which gave similar results.

Expression of curli fimbriae (a major proteinaceous component of the EPS) and cellulose nanofibers (a major polysaccharide component of the EPS) triggers the red, dry, and rough (rdar) morphotype/phenotype on Congo red agar plates (Römling, 2005; Milanov et al., 2015). However, sole expression of cellulose nanofibers leads the pink, dry and rough (pdar) or pink and smooth (pas) morphotype, while sole expression of curli fimbriae creates the brown, dry and rough (bdar) morphotype (Zogaj et al., 2003). In the present study, we observed that all the biofilm producing bacteria produced the rdar morphotype (**Figure 5A**), associated with curli fimbriae and cellulose production. However, intensity of red color, dryness, and roughness of the surfaces were greatly varied in these bacteria (**Figure 5A**). Thus, amount of cellulose and/or curli fimbriae production might be differed in these bacteria.

### Detection of Cellulose by Calcofluor Binding Assays

Because rdar expressing bacteria binds to the cellulose specific dye Calcofluor (Zogaj et al., 2001; Solano et al., 2002; Römling, 2005; Uhlich et al., 2006; Milanov et al., 2015), we therefore evaluated these bacterial strains by spotting the cultures (ca. 10^5^ CFU/mL) on Calcofluor (200 μg/mL) agar plates and incubated at 28°C. After 48 h incubation, Calcofluor agar plates were examined under UV (366 nm) light. However, the fluorescence intensity and pattern were varied greatly in these bacteria (**Figure 5B**). *E. coli* ENSD101 weakly fluoresced only at the side of the colonies, while *E. asburiae* ENSD102, *E. ludwigii* ENSH201, uncultured *Vitreoscilla* sp. ENSG301, *K. pneumoniae* ENSG303, and *P. fluorescens* ENSG304 strongly fluoresced all the spreading zones of the colonies. *A. lwoffii* ENSG302 also strongly fluoresced but covering only 85% of the spreading zones of the colonies, while *B. thuringiensis* ENSW401 fluoresced in a banding pattern. The results of the study confirmed that all these bacteria produced the cellulose-rich polysaccharide.

### Cellulose Production by Different Biofilm Producing Bacteria

**FIGURE 6:**
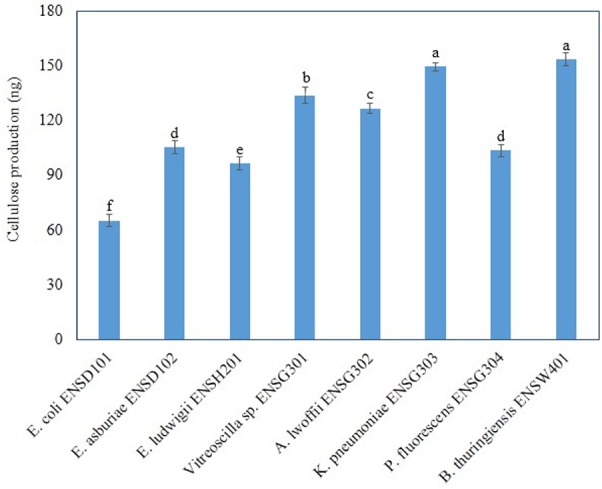
Cellulose production by different biofilm producing bacteria. Cellulose was isolated from 250 mg of lyophilized cell mass obtained from each bacterium grown on Calcofluor agar plates after 48 h incubation at 28°C. The amount of cellulose was determined (OD_620_) by addition of anthrone reagent and Avicel cellulose was used as standard. Error bars represent the standard deviations (±). Values having different letters are significantly different from each other according to Fisher’s least significant difference (LSD) test (*P* ≤ 0.001).

Amount of cellulose production and intensity of fluorescence were correlated in bacteria (Haque et al., 2017), we, therefore, quantified cellulose production in different biofilm producing bacteria grown in Calcofluor agar plates after 48 h incubation at 28°C in static condition. Amount of cellulose production was found to be significantly (*P* ≤ 0.001) varied in these bacteria (**Figure 6**). *B. thuringiensis* ENSW401 produced significantly (*P* ≤ 0.001) more cellulose (153.36 ng), which was followed by *K. pneumoniae* ENSG303 (149.53). The second highest cellulose (133.9 ng) was produced by uncultured *Vitreoscilla* sp. ENSG301. However, cellulose production was statistically similar in *E. asburiae* ENSD102 (105.3 ng) and *P. fluorescens* ENSG304 (103.5 ng). Among the bacteria, *E. coli* ENSD101 produced the lowest amount of cellulose (65.2 ng). Thus, the increase of cellulose production seemed to have been reflected in the increase of Calcofluor binding.

### Bacterial Growth in Response to Different Concentrations of Cu, Zn, Pb, Ni, and Cr

**FIGURE 7:**
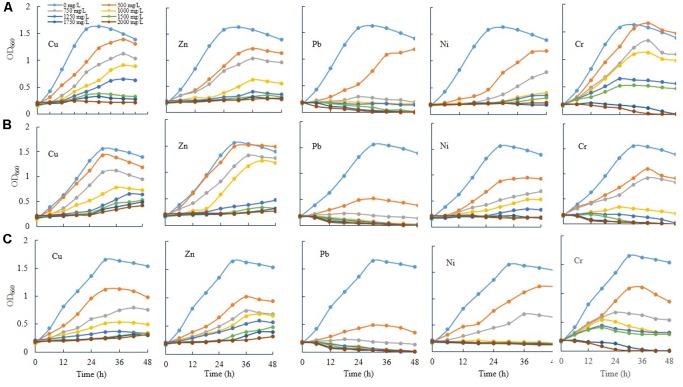
Growth rate of *E. asburiae* ENSD102 **(A)**, uncultured *Vitreoscilla* sp. ENSG301 **(B)**, and *A. lwoffii* ENSG302 **(C)** in shaking condition responding to different concentrations of heavy metals. The data represent one of two separate experiments which gave similar results.

Three novel bacteria, such as *E. asburiae* ENSD102, uncultured *Vitreoscilla* sp. ENSG301, and *A. lwoffii* ENSG302 were tested to ascertain the effect of different concentrations (0, 500, 750, 1000, 1250, 1500, 1750, and 2000 mg/L) of Cu, Zn, Pb, Ni, and Cr on cell growth in shaking condition (**Figure 7**). All the tested bacterial strains grew rapidly in the absence of any heavy metals in SOBG broth. In general, as the concentrations increased, the growth rate was decreased in all the bacteria tested. Among the heavy metals, Pb severely affected the growth. These bacterial strains were incapable to recover their growth exposure to 1750 and 2000 mg/L of Pb and Cr, while they grew only slightly in response to 1750 and 2000 mg/L of Cu and Zn. Thus, specific metals and concentration of the metal might be important for the growth of these bacteria. Interestingly, when 50 μL biofilm cells (10^7^ CFU/mL) of these bacteria (72-h old, biofilm formed on magnesium-deprived SOBG broth containing 500 mg/L of Cu, Zn, Pb, and Cr) were transformed into the glass test tubes containing 5 mL of magnesium-deprived SOBG broth with 1750 and 2000 mg/L of Cu, Zn, Pb, and Cr, the growth was increased in these bacteria in the presence of these metals (data not shown).

### Several Concentrations of Cu, Zn, Pb, Ni, and Cr Stimulates Biofilm Formation

**FIGURE 8:**
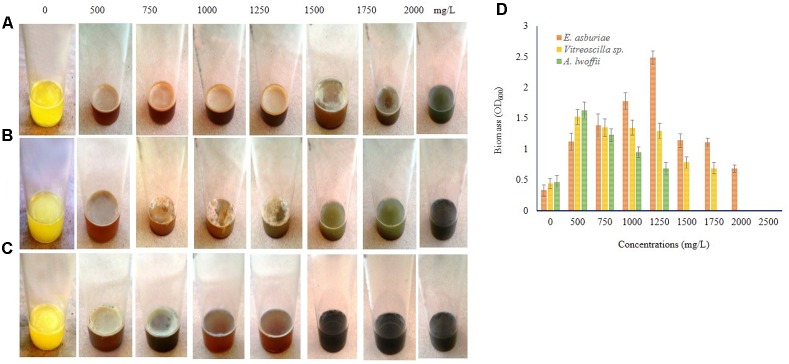
Several concentrations of copper sulfate (for Cu) stimulates biofilm formation in *E. asburiae* ENSD102 **(A)**, uncultured *Vitreoscilla* sp. ENSG301 **(B)**, and *A. lwoffii* ENSG302 **(C)** after 72 h incubation at 28°C in static condition. Biomass of biofilms measured at 600 nm **(D)**. The values are mean and error bars indicate standard deviation (±SD) of the three independent experiments.

Biofilm formation by *E. asburiae* ENSD102, uncultured *Vitreoscilla* sp. ENSG301, and *A. lwoffii* ENSG302 exposure to different concentrations of Cu, Zn, Pb, Ni, and Cr were not studied by any other contemporary researchers yet. *E. asburiae* ENSD102 produced the dense, robust, and smooth AL biofilms in response to 500, 750, and 1250 mg/L of Cu, while they developed the skinny and delicate AL biofilms responding to 1500 and 1750 mg/L of Cu (**Figure 8A**). This bacterium created a faint AL biofilm exposed to 2000 mg/L of Cu (**Figure 8A**). Uncultured *Vitreoscilla* sp. ENSG301 developed a thick, stout, and smooth AL biofilm responding to 500 mg/L of Cu (**Figure 8B**), but they constructed a tinny and uneven AL biofilms responding to 750, 1000, and 1250 mg/L of Cu (**Figure 8B**). Conversely, they generated the weak SAL biofilms in response to 1500 and 1750 mg/L of Cu (**Figure 8B**). Increasing the Cu concentration from 1750 to 2000 mg/L, completely inhibited the biofilm formation in uncultured *Vitreoscilla* sp. ENSG301 (**Figure 8B**). A profuse, firm, and smooth AL biofilm was generated by *A. lwoffii* ENSG302 increasing the Cu concentration from 0 to 500 mg/L (**Figure 8C**), while they developed a thin and fragile AL biofilm in response to 750 mg/L Cu (**Figure 8C**). Conversely, 1000 and 1250 mg/L of Cu triggered the SAL biofilm formation (**Figure 8C**), while 1500 mg/L of Cu prevented the biofilm formation in *A. lwoffii* ENSG302. Nevertheless, the minimal biofilm Cu inhibitory concentration (mg/L) for *E. asburiae* ENSD102, uncultured *Vitreoscilla* sp. ENSG301, and *A. lwoffii* ENSG302 was 2100, 2000, and 1500, respectively. When quantified (**Figure 8D**), compared to the absence of Cu, *E. asburiae* ENSD102 produced 3.39-, 4.61-, 5.4-, 7.53-, 3.38-, 3.37-, and 2.07-fold more biomass biofilms responding to 500, 750, 1000, 1250, 1500, 1750, and 2000 mg/L of Cu, respectively, while uncultured *Vitreoscilla* sp. ENSG301 developed 3.46-, 3.07-, 3.06-, 2.95-, 1.78-, and 1.57-fold higher biomass biofilms responding to 500, 750, 1000, 1250, 1500, and 1750 mg/L of Cu, respectively, and *A. lwoffii* ENSG302 generated 3.47-, 2.62-, 2.03-, and 1.46-fold increase biomass biofilms in response to 500, 750, 1000, and 1250 mg/L of Cu, respectively.

**FIGURE 9:**
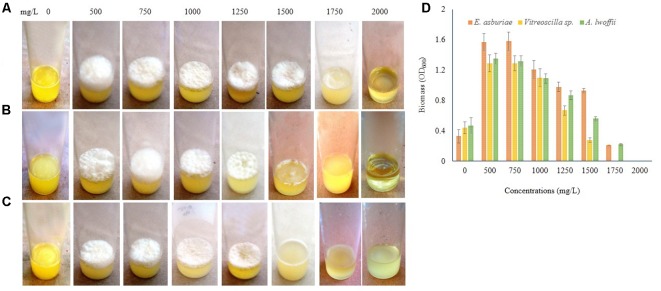
Certain concentrations of zinc sulfate (for Zn) activates biofilm formation in *E. asburiae* ENSD102 **(A)**, uncultured *Vitreoscilla* sp. ENSG301 **(B)**, and *A. lwoffii* ENSG302 **(C)** in static condition after 72 h incubation at 28°C. Biomass of biofilms measured at 600 nm **(D)**. The values are mean and error bars indicate standard deviation (±SD) of the three independent experiments.

Different concentrations of Zn also influenced the biofilm formation (**Figure 9**). *E. asburiae* ENSD102 produced the profuse and rough AL biofilms exposure to 500, 750, 1000, 1250, and 1500 mg/L of Zn (**Figure 9A**), while uncultured *Vitreoscilla* sp. ENSG301 (**Figure 9B**) and *A. lwoffii* ENSG302 (**Figure 9C**) developed the prolific and uneven AL biofilms responding to 500, 750, 1000, and 1250 mg/L of Zn. However, biofilm formation of *E. asburiae* ENSD102 and *A. lwoffii* ENSG302 was prevented by 2000 mg/L of Zn (**Figures 9A,C**), while 1750 mg/L of Zn inhibited the biofilm formation of uncultured *Vitreoscilla* sp. ENSG301 (**Figure 9B**). The minimal biofilm Zn inhibitory concentration (mg/L) for *E. asburiae* ENSD102, uncultured *Vitreoscilla* sp. ENSG301 and *A. lwoffii* ENSG302 was detected at 2000, 2000, and 1750 mg/L, respectively.

**FIGURE 10:**
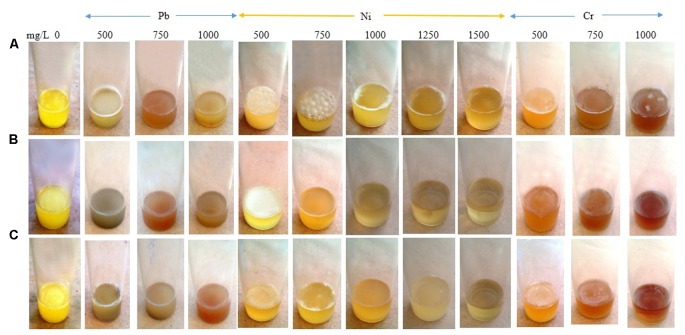
Biofilm formation in static condition by *E. asburiae* ENSD102 **(A)**, uncultured *Vitreoscilla* sp. ENSG301 **(B)**, and *A. lwoffii* ENSG302 **(C)** in response to different concentrations of lead nitrate (for Pb), nickel chloride (for Ni), and potassium dichromate (for Cr) after 72 h incubation at 28°C.

Increasing the Pb concentration from 0 to 500 mg/L triggered an intense SAL biofilm formation by *E. asburiae* ENSD102 and *E. ludwigii* ENSG302, while uncultured *Vitreoscilla* sp. ENSG301 produced a faint SAL biofilm in this concentration (**Figure 10**). *E. asburiae* ENSD102 also induced AL biofilms responding up to 750 mg/L of Ni, while this bacterium generated the weak to strong SAL biofilms increasing the Ni concentration up to 1500 mg/L (**Figure 10**). A stout and thick AL biofilm developed by uncultured *Vitreoscilla* sp. ENSG301 responding to 500 mg/L of Ni, while *A. lwoffii* ENSG302 formed a fragile and thin AL biofilm at this concentration (**Figure 7**). All these bacterial strains also produced a lighter and fragile AL biofilms increasing the Cr concentration up to 750 mg/L (**Figure 10**). Thus, biofilm formation might be dependent on particular metal, concentration of the metal, and bacterial strain. All these bacteria produced the cellulose-rich polysaccharide (**Figures 3**, **4**) responsible for biofilm formation. Polysaccharides were shown to bind with the metals (Ferris et al., 1989; Teitzel and Parsek, 2003; van Hullebusch et al., 2003; Li and Yu, 2014). Thus, these biofilm producing bacterial strains might be an attractive biotechnological tool to remove the toxic heavy metals from wastewaters.

## Discussion

Biofilm formation is an important colonization strategy for adaptation and survival in adverse environmental cues in bacteria. In nature, more than 99% bacteria exists as biofilms (Costerton et al., 1987). Ude et al. (2006) shown that 76% *Pseudomonas* isolates from diverse environmental origins develops AL biofilms on KB broth at 20–22°C within 15 days of incubation in stationary condition in the laboratory. Another survey conducted by Solano et al. (2002), 71% *Salmonella enterica* serovar Enteritidis isolates from environment, food, animals, and clinical origins were found to develop the AL biofilms on Luria-Bertani broth at room temperature in static condition. In this study, only 27.5% (in static condition at 28 and 37°C) and 22.5% (in shaking condition at 28°C) isolates formed the AL and SAL biofilms, respectively, in the glass test tubes containing SOBG broth after 72 h incubation (**Figures 1A,B**) but not in YP, LB, KB, YPDA, and M63 glycerol minimal media (data not shown). SOBG broth was also found as a best biofilm inducing medium by other researchers (Yap et al., 2005; Jahn et al., 2008; Zou et al., 2012; Haque et al., 2009, 2017). Bacterial strains, chemical composition of the surface, nutritional (e.g., media composition, carbon sources, and divalent cations including, magnesium, calcium, and iron), and environmental conditions (e.g., temperature, oxygen tension, osmolarity, pH, and chemotaxis) are important to form the biofilms in the laboratory (Yap et al., 2005; Hossain and Tsuyumu, 2006; Liang et al., 2010; Haque et al., 2012, 2017). Thus, failure to develop the biofilms by several isolates of this study might be due to incongruous nutritional and environmental conditions.

Bacterial biofilm formation depends on production and quantity of EPS (Sutherland, 2001). Concentration and composition of the EPS, hydrodynamic conditions, availability of nutrients, materials of the surface, motility, and intercellular communication system have been shown to regulate biofilm morphology (e.g., smooth and flat, rough, fluffy or filamentous, pillar, and mushroom) in bacteria (Zogaj et al., 2001; Solano et al., 2002; Hall-Stoodley et al., 2004; Flemming and Wingender, 2010). However, in the present study, the isolates of ENSD101, ENSD102, ENSH201, ENSG302, ENSG304, ENSW401, and ENST501 produced the smooth surface AL biofilms, while ENSH202, ENSG301, ENSG303, and ENSG305 developed the rough surface AL biofilms. Thus, biofilm morphology might dependent on bacterial isolates/strains too.

Based on 16S rRNA gene sequencing, *E. coli* (ENSD101 and ENST501), *E. asburiae* (ENSD102), *E. ludwigii* (ENSH201), *P. fluorescens* (ENSH202 and ENSG304), uncultured *Vitreoscilla* sp. (ENSG301 and ENSG305), *A. lwoffii* (ENSG302), *K. pneumoniae* (ENSG303), and *B. thuringiensis* (ENSW401) were identified (**Table 1**). Except uncultured *Vitreoscilla* sp., all these bacteria have been isolated from different wastewaters (Zabłocka-Godlewska et al., 2012; Singh et al., 2015; Khan et al., 2015; Zhi et al., 2016; Radwan et al., 2017; Maintinguer et al., 2017). Importantly, the ability of these bacteria to produce the biofilms in the glass test tubes with SOBG broth was not examined yet. Definitely, certain strains of *E. coli* (Weiss-Muszkat et al., 2010; Hung et al., 2013), *P. fluorescens* (Spiers et al., 2003; Koza et al., 2009), *K. pneumoniae* (Wang et al., 2016), *A. lwoffii* (Martí et al., 2011), and *B. thuringiensis* (El-Khoury et al., 2016) from other than wastewater origins have been reported to form the AL biofilms in defined laboratory systems. In this study, *E. asburiae* ENSD102 (from dyeing wastewater), uncultured *Vitreoscilla* sp. ENSG301 (from garments wastewater) and *E. ludwigii* ENSG302 (from garments wastewater) were identified as novel biofilm producing bacteria.

SEM images results revealed that several matrix of the biofilms produced highly fibrous ribbon-like microfibrils (**Figure 2**), popularly known as cellulose fibrils or nanofibers (Jahn et al., 2011; Hu et al., 2013). XRD data indicated that matrix produced by *E. asburiae* ENSD102, uncultured *Vitreoscilla* sp. ENSG301, and *A. lwoffii* ENSG302 are non-crystalline amorphous in nature (**Figure 4**). These results agree with the previously reported findings (Dogan et al., 2015). The results also came to an agreement that the absence of any ordered crystalline peak is due to the fact that the produced biofilms principally consisted of organic substances without forming any inorganic deposits (Hu et al., 2013). Besides, the literature suggests that presence of protein even in small amount can prevent the crystallization of sugar or sugar–protein mixture (Surewicz and Mantsch, 1988; Sharma and Kalonia, 2004; Haque M.A. et al., 2015). FTIR spectra (**Figure 3**) as well as Congo red binding (**Figure 5A**) results has confirmed that biofilm matrix produced by these bacteria are composed of proteins and cellulose-rich polysaccharides. The component of the matrix in the biofilms of *E. asburiae* ENSD102, uncultured *Vitreoscilla* sp. ENSG301, *A. lwoffii* ENSG302, and *B. thuringiensis* ENSW402 was not reported by any other contemporary researches.

Numerous species from *Enterobacteriaceae* [including certain strains of *E. coli* (Bokranz et al., 2005), some serovars of *Salmonella* (Steenackers et al., 2012), *Enterobacter* sp. (Zogaj et al., 2003; Hungund and Gupta, 2010), *A. baumannii* (Nucleo et al., 2009), *K. pneumoniae* (Zogaj et al., 2003; Wang et al., 2016), and *Pectobacterium carotovorum* subsp. *carotovorum* (Haque et al., 2017)] and *Pseudomonadaceae* [including several species of *Pseudomonas* (Spiers et al., 2003; Ude et al., 2006; Hinsa and O’Toole, 2006) have been reported to produce the cellulose nanofibers and curli fimbriae, the major fraction of the EPS matrix. It was reported that expression of cellulose nanofibers and/or curli fimbriae depend on bacterial species/strains, chemical composition of the surfaces, growth, and environmental conditions (Prouty and Gunn, 2003; Gerstel and Römling, 2003; García et al., 2004; Yap et al., 2005; Haque et al., 2012, 2017; Haque M.M. et al., 2015). EPS not only contains cellulose nanofibers and curli fimbriae but also contain extracellular DNA (Whitchurch et al., 2002; Liang et al., 2010). In this study, when we added up to 1000 U/mL of DNase I to SOBG broth during biofilm formation process by these bacteria and incubated the culture at 28°C in static condition, all these bacteria produced the AL biofilms after 72 h incubation (data not shown). Thus, EPS of these bacterial strains might not be contained the extracellular DNA.

Bacterial biofilms are resistant to toxic metal ions (Teitzel and Parsek, 2003; Harrison et al., 2005; Koechler et al., 2015). Certain metal ions, such as Ca^2+^, Mg^2+^, Fe^2+^, Fe^3+^, Ba^2+^, Cu^2+^, and Zn^2+^ induced the biofilm formation in bacteria (Turakhia and Characklis, 1989; Rinaudi et al., 2006; Song and Leff, 2006; Liang et al., 2010; Haque et al., 2012). Not only metals but their concentrations also played an important role in biofilm formation in bacteria. For example, increasing the Cu^2+^ concentration from 50 to 100 μM increased the biofilm formation in *Xylella fastidiosa* strain Temecula, while higher concentrations (>200 μM) prevented the biofilm formation (Cobine et al., 2013). *X. fastidiosa* also increased the biofilm formation when PD2 amended with 400 μM ZnSO_4_ under flow conditions and with constant bacterial feeding (Navarrete and De La Fuente, 2014). *E. coli* K-12 produced twofold more biofilm biomass in the presence of 100 μM of nickel compared to the biofilm grown in the absence of this metal (Perrin et al., 2009). We observed that several concentrations of Cu, Zn, Pb, Ni, and Cr stimulated the biofilm formation (**Figures 8**–**10**). We do not know exactly why several concentrations of these metals increased the biofilm formation in *E. asburiae* ENSD102, uncultured *Vitreoscilla* sp. ENSG301, and *A. lwoffii* ENSG302. Current study showed that all these bacteria produced both proteinaceous curli fimbriae and cellulose-rich polysaccharide (**Figures 4**, **5**). The protein units reportedly gave the characteristics IR band through C = O stretching at amide I region, N–H bending and C–N stretching at amide II region and C–N bending and N–H stretching at amide III region (Liaqat et al., 2009; Haque et al., 2014). On the other hand, band region for polysaccharide principally resulted by stretching vibration of C–C and C–O bonds and deformation of C–O–H and C–O–C bonds (Naumann, 2000; Grube et al., 2002). It was reported that the positively charged metal bound with negatively charged functional groups present on the bacteria (Teitzel and Parsek, 2003; van Hullebusch et al., 2003). Thus, protein and/or polysaccharide produced by *E. asburiae* ENSD102, uncultured *Vitreoscilla* sp. ENSG301, and *A. lwoffii* ENSG302 in response to different concentrations of Cu, Zn, Pb, Ni, and Cr could sequester the toxic metal ions, giving to the bacteria the time required for adaptation thus driving to the physiological or metabolic changes necessary for eliminating the toxic effect of these metals, i.e., expression of enzymes and transporters for pumping out the metal or metal-binding proteins (Letelier et al., 2010; Mindlin et al., 2016; Nocelli et al., 2016; Karn et al., 2017). Thus, these bacterial strains might be an attractive biotechnological tool for bioremediation of toxic heavy metals from wastewaters. Recently, several researchers have been shown that heavy metal resistant bacteria were also multidrug resistant (Bhagat et al., 2016; Aransiola et al., 2017; Andrade et al., 2018). Therefore, future studies should focus on study the virulence factor of these bacteria before used in bioremediation of heavy metals.

## Conclusion

Eleven biofilm producing bacterial strains were isolated and identified from diverse wastewaters of Bangladesh using 16S rRNA gene sequencing. All these bacteria produced proteinaceous curli fimbriae and cellulose—the two major components of the EPS. Cellulose has a wide variety of biomedical applications (e.g., wound dressing and blood vessels) as well as tissue engineering fields. Bacterial growth rate was decreased with the increase of the concentrations of the Cu, Zn, Pb, Ni, and Cr. Several concentrations of these heavy metals significantly enhanced the biofilm formation in *E. asburiae* ENSD102, uncultured *Vitreoscilla* sp. ENSG301, and *A. lwoffii* ENSG302. Biofilm/EPS matrix act as molecular sieve, e.g., sequestering metal ions, these bacterial strains might be an attractive biotechnological tool for bioremediation of Cu, Zn, Cr, Ni, and Pb from wastewaters.

## Author Contributions

MM, ZHT, and MMH conducted the experiments. MMH conceived the idea, wrote the manuscript, and collected the research fund. MK, AM, MA, and MRT characterized the isolates and analyzed the data. MI identified the bacteria based on 16S rRNA gene sequencing. MAH conducted the FTIR analysis of the matrix of the biofilms produced by different bacteria. All the authors read the manuscript and approved for the submission.

## Conflict of Interest Statement

The authors declare that the research was conducted in the absence of any commercial or financial relationships that could be construed as a potential conflict of interest.

## Footnotes

1 http://www.ncbi.nlm.nih.gov/

## References

[B1] Ahluwalia S. S. Goyal D. (2007). Microbial and plant derived biomass for removal of heavy metals from wastewater. *Bioresour. Technol.* 98 2243–2257. 10.1016/j.biortech.2005.12.006 16427277

[B2] Ahmed M. K. Baki M. A. Kundu G. K. Islam M. S. Islam M. M. Hossain M. M. (2016). Human health risks from heavy metals in fish of Buriganga river, Bangladesh. *Springer Plus* 5:1697. 10.1186/s40064-016-3357-0 27757369 PMC5047865

[B3] Alam M. Z. Carpenter-Boggs L. Rahman A. Haque M. M. Miah M. R. U. Moniruzzaman M. (2017). Water quality and resident perceptions of declining ecosystem services at Shitalakka wetland in Narayanganj city. *Sustain. Water Qual. Ecol.* 9–10, 53–66. 10.1016/j.swaqe.2017.03.002

[B4] Al-Awadhi H. Al-Hasan R. H. Sorkhoh N. A. Salamah S. Radwan S. S. (2003). Establishing oil-degrading biofilms on gravel particles and glass plates. *Int. Biodeterior. Biodegradation* 51 181–185. 10.1016/S0964-8305(02)00140-3

[B5] American Public Health Association [APHA] (1998). *Standard Methods for the Examination of Water and Wastewater*, 20th Edn Washington, DC: American Public Health Association.

[B6] Andrade L. N. Siqueira T. E. S. Martinez R. Darini A. L. C. (2018). Multidrug-resistant CTX-M-(15, 9,2)- and KPC-2-producing *Enterobacter hormaechei* and *Enterobacter asburiae* isolates possessed a set of acquired heavy metal tolerance genes including a chromosomal *sil* operon (for acquired silver resistance). *Front. Microbiol.* 9:539. 10.3389/fmicb.2018.00539 29628916 PMC5876308

[B7] Aransiola E. F. Ige O. A. Ehinmitola E. O. Layokun S. K. (2017). Heavy metals bioremediation potential of *Klebsiella* species isolated from diesel polluted soil. *Afr. J. Biotechnol.* 16 1098–1105. 10.5897/AJB2016.15823 24912107

[B8] Bhagat N. Vermani M. Bajwa H. S. (2016). Characterization of heavy metal (cadmium and nickle) tolerant Gram negative enteric bacteria from polluted Yamuna River, Delhi. *Afr. J. Microbiol. Res.* 10 127–137. 10.5897/AJMR2015.7769

[B9] Bokranz W. Wang X. Tschape H. Romling U. (2005). Expression of cellulose and curli fimbriae by *Escherichia coli* isolated from the gastrointestinal tract. *J. Med. Microbiol.* 54 1171–1182. 10.1099/jmm.0.46064-0 16278431

[B10] Castresana J. (2000). Selection of conserved blocks from multiple alignments for their use in phylogenetic analysis. *Mol. Biol. Evol.* 17 540–552. 10.1093/oxfordjournals.molbev.a026334 10742046

[B11] Chang W. C. Hsu G. S. Chiang S. M. Su M. C. (2006). Heavy metal removal from aqueous solution by wasted biomass from a combined AS-biofilm process. *Bioresour. Technol.* 97 1503–1508. 10.1016/j.biortech.2005.06.011 16112569

[B12] Cobine P. A. Cruz L. F. Navarrete F. Duncan D. Tygart M. De Le Fuente L. (2013). *Xylella fastidiosa* differentially accumulates mineral elements in biofilm and planktonic cells. *PLoS One* 8:e54936. 10.1371/journal.pone.0054936 23349991 PMC3551809

[B13] Conrad A. Suutari M. K. Keinänen M. M. Cadoret A. Faure P. Mansuy-Huault L. (2003). Fatty acid lipid fractions in extracellular polymeric substances of activated sludge flocs. *Lipids* 38 1093–1105. 10.1007/s11745-006-1165-y 14669975

[B14] Costerton J. W. Cheng K. J. Geesey G. G. Ladd T. I. Nickel J. C. Dasgupta M. (1987). Bacterial biofilms in nature and disease. *Annu. Rev. Microbiol.* 41 435–464. 10.1146/annurev.mi.41.100187.002251 3318676

[B15] Costerton J. W. Stewart P. S. Greenberg E. P. (1999). Bacterial biofilms: a common cause of persistent infections. *Science* 284 1318–1322. 10.1126/science.284.5418.1318 10334980

[B16] Cristina Q. Zelia R. Bruna F. Hugo F. Teresa T. (2009). Biosorptive performance of an *Escherichia coli* biofilm supported on zeolite NaY for the removal of Cr(VI), Cd(II), Fe(III) and Ni(II). *Chem. Eng. J.* 152 110–115. 10.1016/j.cej.2009.03.039

[B17] Das N. Basak L. V. G. Salam J. A. Abigail M. E. A. (2012). Application of biofilms on remediation of pollutants – an overview. *J. Microbiol. Biotechnol. Res.* 2 783–790. 10.1007/s00253-013-5216-z 24150788

[B18] Das P. Aziz S. Obbard J. (2011). Two phase microalgae growth in the open system for enhanced lipid productivity. *Renew. Energy* 36 2524–2528. 10.1016/j.renene.2011.02.002

[B19] Davey M. E. Cajazza N. C. O’Toole G. A. (2003). Rhamnolipid surfactant production affects biofilm architecture in *Pseudomonas aeruginosa* PAO1. *J. Bacteriol.* 185 1027–1036. 10.1128/JB.185.3.1027-1036.2003 12533479 PMC142794

[B20] Dixit R. Wasiulla M. D. Pandiyan K. Singh U. B. Sanu A. (2015). Bioremediation of heavy metals from soil and aquatic environment: an overview of principles and criteria of fundamental processes. *Sustainability* 7 2189–2212. 10.3390/su7022189

[B21] Dogan N. M. Doganli G. A. Dogan G. Bozkaya O. (2015). Characterization of extracellular polysaccharide (EPS) produced by thermal *Bacillus* and determination of environmental conditions affecting exopolysaccharide production. *Int. J. Environ. Res.* 9 1107–1116. 10.22059/IJER.2015.998

[B22] Donlan R. M. Costerton J. W. (2002). Biofilms: survival mechanisms of clinically relevant microorganisms. *Clin. Microbiol. Rev.* 15 167–193. 10.1128/CMR.15.2.167-193.2002 11932229 PMC118068

[B23] Edgar R. C. (2004). MUSCLE: multiple sequence alignment with high accuracy and high throughput. *Nucleic Acids Res.* 32 1792–1797. 10.1093/nar/gkh340 15034147 PMC390337

[B24] Edwards S. J. Kjellerup B. V. (2013). Applications of biofilms in bioremediation and biotransformation of persistent organic pollutants, pharmaceutical/personal care products, and heavy metals. *Appl. Microbiol. Biotechnol.* 97 9909–9921. 10.1007/s00253-013-5216-z 24150788

[B25] Elekwachi C. O. Andresen J. Hodgman T. C. (2014). Global use of bioremediation technologies for decontamination of ecosystems. *J. Bioremediat. Biodegrad.* 5 1–9. 10.4172/2155-6199.1000225 16758707

[B26] El-Khoury N. Majed R. Perchat S. Kallassy M. Lereclus D. Gohar M. (2016). Spatio-temporal evolution of sporulation in *Bacillus thuringiensis* biofilm. *Front. Microbiol.* 7:1222. 10.3389/fmicb.2016.01222 27536298 PMC4971082

[B27] Fang L. Wei X. Cai P. Huang Q. Chen H. Liang W. (2011). Role of extracellular polymeric substances in Cu(II) adsorption on *Bacillus subtilis* and *Pseudomonas putida*. *Bioresour. Technol.* 102 1137–1141. 10.1016/j.biortech.2010.09.006 20869870

[B28] Ferris F. G. Schultze S. Witten T. C. Fyfe W. S. Beveridge T. J. (1989). Metal interactions with microbial biofilms in acidic and neutral pH environments. *Appl. Environ. Microbiol.* 55 1249–1257. 16347914 10.1128/aem.55.5.1249-1257.1989 PMC184285

[B29] Fida T. T. Breugelmans P. Lavigne R. Coronado E. Johnson D. R. vander Meer J. R. (2012). Exposure to solute stress affects genome-wide expression but not the poly-cyclic aromatic hydrocarbon-degrading activity of *Sphingomonas* sp. strain LH128 in biofilms. *Appl. Environ. Microbiol.* 78 8311–8320. 10.1128/AEM.02516-12 23001650 PMC3497376

[B30] Flemming H. C. Wingender J. (2010). The biofilm matrix. *Nat. Rev. Microbiol.* 8 623–633. 10.1038/nrmicro2415 20676145

[B31] García B. Latasa C. Solano C. García-del Portillo F. Gamazo C. Lasa I. (2004). Role of the GGDEF protein family in *Salmonella* cellulose biosynthesis and biofilm formation. *Mol. Microbiol.* 54 264–277. 10.1111/j.1365-2958.2004.04269.x 15458421

[B32] Gerstel U. Römling U. (2003). The *csgD* promoter, a control unit for biofilm formation in *Salmonella typhimurium*. *Res. Microbiol.* 154 659–667. 10.1016/j.resmic.2003.08.005 14643403

[B33] Grube M. Bekers M. Upite D. Kaminska E. (2002). Infrared spectra of some fructans. *Spectroscopy* 16 289–296. 10.1155/2002/637587

[B34] Hall-Stoodley L. Costerton J. W. Stoodley P. (2004). Bacterial biofilms: from the natural environment to infectious diseases. *Nat. Rev. Microbiol.* 2 95–108. 10.1038/nrmicro821 15040259

[B35] Haque M. A. Aldred P. Chen J. Adhikari B. (2015). Denaturation and physical characteristics of spray dried whey protein isolate powders produced in the presence and absence of lactose, trehalose and polysorbate- 80. *Drying Technol.* 33 1243–1254. 10.1080/07373937.2015.1023311

[B36] Haque M. A. Aldred P. Chen J. Barrow C. J. Adhikari B. (2014). Drying and denaturation characteristics of α-lactalbumin, β-lactoglobulin and bovine serum albumin in convective drying process. *J. Agric. Food Chem.* 62 4695–4706. 10.1021/jf405603c 24819828

[B37] Haque M. M. Hirata H. Tsuyumu S. (2012). Role of PhoP-PhoQ two-component system in pellicle formation, virulence and survival in harsh environments of *Dickeya dadantii* 3937. *J. Gen. Plant Pathol.* 78 176–189. 10.1007/s10327-012-0372-z

[B38] Haque M. M. Hirata H. Tsuyumu S. (2015). SlyA regulates motA and motB, virulence and stress-related genes under conditions induced by the PhoP-PhoQ system in *Dickeya dadantii* 3937. *Res. Microbiol.* 166 467–475. 10.1016/j.resmic.2015.05.004 26027774

[B39] Haque M. M. Kabir M. S. Aini L. Q. Hirata H. Tsuyumu S. (2009). SlyA, a MarR family transcriptional regulator, is essential for virulence in *Dickeya dadantii* 3937. *J. Bacteriol.* 191 5409–5419. 10.1128/JB.00240-09 19542281 PMC2725626

[B40] Haque M. M. Oliver M. M. H. Nahar K. Alam M. Z. Hirata H. Tsuyumu S. (2017). CytR homolog of *Pectobacterium carotovorum* subsp. *carotovorum* controls air-liquid biofilm formation by regulating multiple genes involved in cellulose production, c-di-GMP signaling, motility, and type III secretion system in response to nutritional and environmental signals. *Front. Microbiol.* 8:972. 10.3389/fmicb.2017.00972 28620360 PMC5449439

[B41] Harrison J. J. Turner R. J. Ceri H. (2005). Persister cells, the biofilm matrix and tolerance to metal cations in biofilm and planktonic *Pseudomonas aeruginosa*. *Environ. Microbiol.* 7 981–994. 10.1111/j.1462-2920.2005.00777.x 15946294

[B42] Hinsa S. M. O’Toole G. A. (2006). Biofilm formation by *Pseudomonas fluorescens* WCS365: a role for LapD. *Microbiology* 152 1375–1383. 10.1099/mic.0.28696-0 16622054

[B43] Hossain M. M. Tsuyumu S. (2006). Flagella-mediated motility is required for biofilm formation by *Erwinia carotovora* subsp. *carotovora*. *J. Gen. Plant Pathol.* 72 34–39. 10.1007/s10327-005-0246-8

[B44] Hu X.-B. Xu K. Wang Z. Ding L.-L. Ren H.-Q. (2013). Characteristics of biofilm attaching to carriers in moving bed biofilm reactor used to treat vitamin C wastewater. *Scanning* 35 283–291. 10.1002/sca.21064 23168685

[B45] Huang Y.-B. Wang W.-H. Pemg A. (2000). Accumulation of Cu(II) and Pb(II) by biofilms grown on particulate in aquatic systems. *J. Environ. Sci. Health Part A Environ. Sci. Eng.* 35 575–592. 10.1080/10934520009376987

[B46] Hung C. Zhou Y. Pinkner J. S. Dodson K. W. Crowley J. R. Heuser J. (2013). *Escherichia coli* biofilms have an organized and complex extracellular matrix structure. *mBio* 4:e00645–13. 10.1128/mBio.00645-13 24023384 PMC3774191

[B47] Hungund B. S. Gupta S. G. (2010). Improved production of bacterial cellulose from *Gluconacetobacter persimmonis* GH-2. *J. Microb. Biochem. Technol.* 2 127–133. 10.4172/1948-5948.1000037

[B48] Islam M. M. Mahmud K. Faruk O. Billah M. S. (2011). Textile dyeing industries in Bangladesh for sustainable development. *Int. J. Environ. Sci. Dev.* 2 428–436. 10.7763/IJESD.2011.V2.164

[B49] Islam M. S. Ahmed M. K. Habibullah-Al-Mamun M. (2014). Determination of heavy metals in fish and vegetables in Bangladesh and health implications. *Hum. Ecol. Risk Assess. Int. J.* 21 986–1006. 10.1080/10807039.2014.950172

[B50] Islam M. S. Ahmed M. K. Raknuzzaman M. Habibullah-Al-Mamun M. Masunaga S. (2015). Metal speciation in sediment and their bioaccumulation in fish species of three urban rivers in Bangladesh. *Arch. Environ. Contam. Toxicol.* 68 92–106. 10.1007/s00244-014-0079-6 25213477

[B51] Jahn C. E. Selimi D. A. Barak J. D. Charkowski A. O. (2011). The Dickeya dadantii biofilm matrix consists of cellulose nanofibres, and is an emergent property dependent upon the type III secretion system and the cellulose synthesis operon. *Microbiology* 157 2733–2744. 10.1099/mic.0.051003-0 21719543

[B52] Jahn C. E. Willis D. K. Charkowski A. O. (2008). The flagellar sigma factor FliA is required for *Dickeya dadantii* virulence. *Mol. Plant Microbe Interact.* 11 1431–1442. 10.1094/MPMI-21-11-1431 18842093

[B53] Jin H. Liu G. Tao W. (2007). Decolourization of a dye industry effluent by *Aspergillus fumigatus* XC6. *Appl. Microbiol. Biotechnol.* 74 239–243. 10.1007/s00253-006-0658-1 17086413

[B54] Jones D. T. Taylor W. R. Thornton J. M. (1992). The rapid generation of mutation data matrices from protein sequences. *Comput. Appl. Biosci.* 8 275–282. 10.1093/bioinformatics/8.3.275 1633570

[B55] Kaplan J. B. (2010). Biofilm dispersal: mechanisms, clinical implications, and potential therapeutic uses. *J. Dent. Res.* 89 205–218. 10.1177/0022034509359403 20139339 PMC3318030

[B56] Karn S. K. Fang G. Duan J. (2017). *Bacillus* sp. acting as dual role for corrosion induction and corrosion inhibition with carbon steel (CS). *Front. Microbiol.* 8:2038. 10.3389/fmicb.2017.02038 29114242 PMC5660695

[B57] Khan Z. Hussain S. Z. Rehman A. Zulfiqar S. Shakoori A. R. (2015). Evaluation of cadmium resistant bacterium, *Klebsiella pneumoniae*, isolated from industrial wastewater for its potential use to bioremediate environmental cadmium. *Pak. J. Zool.* 47 1533–1543.

[B58] Klemm D. Schumann D. Udhardt U. Marsch S. (2001). Bacterial synthesized cellulose-artificial blood vessels for microsurgery. *Prog. Polym. Sci.* 26 1561–1603. 10.1016/S0079-6700(01)00021-1

[B59] Koechler S. Farasin J. Cleiss-Arnold J. Arséne-Ploetze F. (2015). Toxic metal resistance in biofilms: diversity of microbial responses and their evolution. *Res. Microbiol.* 10 764–773. 10.1016/j.resmic.2015.03.008 25869223

[B60] Koza A. Hallett P. D. Moon C. D. Spiers A. J. (2009). Characterization of a novel air-liquid interface biofilm of *Pseudomonas fluorescens* SBW25. *Microbiology* 155 1397–1406. 10.1099/mic.0.025064-0 19383709

[B61] Labrenz M. Druschel G. K. Thomsen-Ebert T. Gilbert B. Welch S. A. Kemner K. M. (2000). Formation of sphalerite (ZnS) deposits in natural biofilms of surface-reducing bacteria. *Science* 290 1744–1747. 10.1126/science.290.5497.1744 11099408

[B62] Lasa I. Penadé J. R. (2006). Bap: a family of surface proteins involved in biofilm formation. *Res. Microbiol.* 157 99–107. 10.1016/j.resmic.2005.11.003 16427771

[B63] Letelier M. E. Sebastian S. J. Liliana P. S. Cortés-Troncoso J. Aracena-Parks P. (2010). Mechanisms underlying iron and copper ions toxicity in biological systems: pro-oxidant activity and protein-binding effects. *Chem. Biol. Interact.* 188 220–227. 10.1016/j.cbi.2010.06.013 20603110

[B64] Li W.-W. Yu H.-Q. (2014). Insight into the roles of microbial extracellular polymer substances in metal biosorption. *Bioresour. Technol.* 160 15–23. 10.1016/j.biortech.2013.11.074 24345430

[B65] Liang Y. Gao H. Chen J. Dong Y. Wu L. He Z. (2010). Pellicle formation in *Shewanella oneidensis*. *BMC Microbiol.* 10:291. 10.1186/1471-2180-10-291 21080927 PMC2995470

[B66] Liaqat I. Sumbal F. Sabri A. N. (2009). Tetracycline and chloramphenicol efficiency against selected biofilm forming bacteria. *Curr. Microbiol.* 59 212–220. 10.1007/s00284-009-9424-9 19484302

[B67] Maintinguer S. I. Lazaro C. Z. Pachiega R. Varesche M. B. A. Sequinel R. Oliveira J. E. (2017). Hydrogen bioproduction with *Enterobacter* sp. isolated from brewery wastewater. *Int. J. Hydrogen Energy* 42 152–160. 10.1016/j.ijhydene.2016.11.104

[B68] Maneerung T. Tokura S. Rujiravanit R. (2007). Impregnation of silver nanoparticles into bacterial cellulose for antimicrobial wound dressing. *Carbohydr. Polym.* 72 43–51. 10.1016/j.carbpol.2007.07.025

[B69] Martí S. Rodríguez-Baño J. Catel-Ferreira M. Jouenne T. Vila J. Seifert H. (2011). Biofilm formation at the solid-liquid and air-liquid interfaces by *Acinetobacter* species. *BMC Short Notes* 4:5. 10.1186/1756-0500-4-5 21223561 PMC3023692

[B70] Martín-Cereceda M. Jorand F. Guinea A. Block J. C. (2001). Characterization of extracellular polymeric substances in rotating biological contactors and activated sludge flocs. *J. Environ. Technol.* 22 951–959. 10.1080/09593332208618231 11561952

[B71] McDougald D. Rice S. A. Barraud N. Steinberg P. D. Kjelleberg S. (2012). Should we stay or should we go: mechanisms and ecological consequences for biofilm dispersal. *Nat. Rev. Microbiol.* 10 39–50. 10.1038/nrmicro2695 22120588

[B72] Milanov D. S. Prunic B. Z. Velhner M. J. Pajic M. L. Cabarkapa I. S. (2015). Rdar morphotype- a resting stage of some *Enterobacteriaceae*. *Food Feed Res.* 42 43–50. 10.5937/FFR1501043M

[B73] Mindlin S. Petrenko A. Kurakov A. Beletsky A. Mardanov A. Petrova M. (2016). Resistance of permafrost and modern *Acinetobacter lwoffii* strains to heavy metals and arsenic revealed by genome analysis. *BioMed Res. Int.* 2016:3970831. 10.1155/2016/3970831 27795957 PMC5067307

[B74] Mitra A. Mukhopadhyay S. (2016). Biofilm mediated decontamination of pollutants from the environment. *AIMS Bioeng.* 3 44–59. 10.3934/bioeng.2016.1.44

[B75] Muñoz R. Alvarez M. T. Muñoz A. Terrazas E. Guieysse B. Mattiasson B. (2006). Sequential removal of heavy metals ions and organic pollutants using an algal680 bacterial consortium. *Chemosphere* 63 903–911. 10.1016/j.chemosphere.2005.09.062 16307789

[B76] Naser H. M. Sultana S. Haque M. M. Akhter S. Begum R. A. (2014). Lead, cadmium and nickel accumulation in some common spice grown in industrial areas of Bangladesh. *Agriculturists* 12 122–130. 10.3329/agric.v12i1.19867

[B77] Naumann D. (2000). “FT-Infrared and FT-Raman spectroscopy in biomedical research,” in *Infrared and Raman Spectroscopy of Biological Materials*, eds Gremlich H. U. Yan B. (Basel: Marcel Dekker, Inc.), 323–377. 10.1021/ja004845m

[B78] Navarrete F. De La Fuente L. (2014). Response of *Xylella fastidiosa* to zinc: decreased culturability, increased exopolysaccharide production, and formation of resilient biofilms under flow conditions. *Appl. Environ. Microbiol.* 80 1097–1107. 10.1128/AEM.02998-13 24271184 PMC3911211

[B79] Nocelli N. Bogino P. C. Banchio E. Giordano W. (2016). Roles of extracellular polysaccharides and biofilm formation in heavy metal resistance of rhizobia. *Materials* 9:418. 10.3390/ma9060418 28773540 PMC5456807

[B80] Nucleo E. Steffanoni L. Fugazza G. Migliavacca R. Giacobone E. Navarra A. (2009). Growth in glucose-based medium and exposure to subinhibitory concentrations of imipenem induce biofilm formation in a multidrug-resistant clinical isolate of *Acinetobacter baumannii*. *BMC Microbiol.* 9:270. 10.1186/1471-2180-9-270 20028528 PMC2804601

[B81] Pal A. Paul A. K. (2008). Microbial extracellular polymeric substances: central elements in heavy metal bioremediation. *Indian J. Microbiol.* 48 49–64. 10.1007/s12088-008-0006-5 23100700 PMC3450203

[B82] Payne R. B. May H. D. Sowers K. B. (2011). Enhanced reductive dechlorination of polychlorinated biphenyl impacted sediment by bioaugmentation with a dehalorespiring bacterium. *Environ. Sci. Technol.* 45 8772–8779. 10.1021/es201553c 21902247 PMC3210572

[B83] Perrin C. Briandet R. Jubelin G. Lejeume P. Mandrand-Berthelot M. A. Rodrigue A. (2009). Nickel promotes biofilm formation by *Escherichia coli* K-12 strains that produce curli. *Appl. Environ. Microbiol.* 75 1723–1733. 10.1128/AEM.02171-08 19168650 PMC2655473

[B84] Prigent-Combaret C. Prensier G. Le Thi T. T. Vidal O. Lejeune P. Dorel C. (2000). Development pathway for biofilm formation in curli-producing *Escherichia coli* strains: roles for flagella, curli and colonic acid. *Environ. Microbiol.* 2 450–464. 10.1046/j.1462-2920.2000.00128.x 11234933

[B85] Prouty A. M. Gunn J. S. (2003). Comparative analysis of *Salmonella enterica* serovar Typhimurium biofilm formation on gallstones and glass. *Infect. Immun.* 71 7154–7158. 10.1128/IAI.71.12.7154-7158.2003 14638807 PMC308894

[B86] Radwan T. E. E. Reyad A. M. M. Essa A. M. M. (2017). Bioremediation of the nematicide oxamyl by *Enterobacter ludwigii* isolated from agricultural wastewater. *Egypt. J. Exp. Biol.* 13 19–30. 10.5455/egyjebb.20170131064321

[B87] Rinaudi L. Fujishige N. A. Hirsch A. M. Banchio E. Zorreguieta A. Giordano W. (2006). Effects of nutritional and environmental conditions on *Sinorhizobium meliloti* biofilm formation. *Res. Microbiol.* 15 867–875. 10.1016/j.resmic.2006.06.002 16887339

[B88] Römling U. (2005). Characterization of the rdar morphotype, a multicellular behavior in *Enterobacteriaceae*. *Cell Mol. Life Sci.* 62 1234–1246. 10.1007/s00018-005-4557-x 15818467 PMC11139082

[B89] Römling U. Galperin M. (2015). Bacterial cellulose biosynthesis: diversity, of operons, subunits, products and functions. *Trends Microbiol.* 23 545–557. 10.1016/j.tim.2015.05.005 26077867 PMC4676712

[B90] Sambrook J. Fritsch E. F. Maniatis T. (1989). *Molecular Cloning*, 2nd Edn Cold Spring Harbor, NY: Cold Spring Harbor Laboratory Press.

[B91] Saratale R. G. Saratale G. D. Chang J. S. Govindwar S. P. (2011). Bacterial decolorization and degradation of azo dyes: a review. *J. Taiwan Inst. Chem. Eng.* 42 138–157. 10.1016/j.jtice.2010.06.006 25665634

[B92] Seo Y. Lee W. H. Sorial G. Bishop P. L. (2009). The application of a mulch biofilm barrier for surfactant enhanced polycyclic aeromatic hydrocarbon bioremediation. *Environ. Pollut.* 157 95–101. 10.1016/j.envpol.2008.07.022 18973969

[B93] Sharma V. K. Kalonia D. S. (2004). Effect of vacuum drying on protein-mannitol interactions: the physical state of mannitol and protein structure in the dried state. *AAPS PharmSciTech* 5 1–12. 10.1208/pt050110 15198531 PMC2784861

[B94] Sheikh A. H. Molla A. H. Haque M. M. Hoque M. Z. Alam M. Z. (2017). Evaluation of water quality and biodiversity of natural freshwater wetlands discharged by industrial effluent. *Acad. J. Environ. Sci.* 5 52–64. 10.15413/ajes.2017.0123

[B95] Singh A. L. Chaudhary S. Kayastha A. M. Yadav A. (2015). Decolorization and degradation of textile effluent with the help of *Enterobacter asburiae*. *Indian J. Biotechnol.* 14 101–106.

[B96] Singh R. Paul D. Jain R. K. (2006). Biofilms: implications in bioremediation. *Trends Microbiol.* 14 389–397. 10.1016/j.tim.2006.07.001 16857359

[B97] Solano C. García B. Valle J. Berasain C. Ghigo J. M. Gamazo C. (2002). Genetic analysis of *Salmonella enteritidis* biofilm formation: critical role of cellulose. *Mol. Microbiol.* 43 793–808. 10.1046/j.1365-2958.2002.02802.x 11929533

[B98] Song B. Leff L. G. (2006). Influence of magnesium ions on biofilm formation by *Pseudomonas fluorescens*. *Microbiol. Res.* 161 355–361. 10.1016/j.micres.2006.01.004 16517137

[B99] Spiers A. J. Bohannon J. Gehrig S. M. Rainey P. B. (2003). Biofilm formation at the air-liquid interface by the *Pseudomonas fluorescens* SBW25 wrinkly spreader requires an acetylated for of cellulose. *Mol. Microbiol.* 50 15–27. 10.1046/j.1365-2958.2003.03670.x 14507360

[B100] Steenackers H. Hermans K. Vanderleyden J. Keersmaecker D. (2012). *Salmonella* biofilms: an overview on occurrence, structure, regulation and eradication. *Food Res. Int.* 45 502–531. 10.1016/j.foodres.2011.01.038

[B101] Sundar K. Sadiq M. Mukherjee A. Chandrasekaran N. (2011). Bioremoval of trivalent chromium using *Bacillus* biofilms through continuous flow reactor. *J. Hazard. Mater.* 741 44–51. 10.1016/j.jhazmat.2011.08.066 21924829

[B102] Surewicz W. K. Mantsch H. H. (1988). New insight into protein secondary structure from resolution-enhanced infrared spectra. *Biochim. Biophys. Acta* 952 115–130. 10.1016/0167-4838(88)90107-0 3276352

[B103] Sutherland I. W. (2001). The biofilm matrix – an immobilized but dynamic microbial environment. *Trends Microbiol.* 9 222–227. 10.1016/S0966-842X(01)02012-1 11336839

[B104] Tamura K. Stecher G. Peterson D. Filipski A. Kumar S. (2013). MEGA6: molecular evolutionary genetics analysis version 6.0. *Mol. Biol. Evol.* 30 2725–2729. 10.1093/molbev/mst197 24132122 PMC3840312

[B105] Teitzel G. M. Parsek M. R. (2003). Heavy metal resistance of biofilm and planktonic *Pseudomonas aeruginosa*. *Appl. Environ. Microbiol.* 69 2313–2320. 10.1128/AEM.69.4.2313-2320.2003 12676715 PMC154819

[B106] Turakhia M. H. Characklis W. G. (1989). Activity of *Pseudomonas aeruginosa* in biofilms-effect of calcium. *Biotechnol. Bioeng.* 33 406–414. 10.1002/bit.260330405 18587931

[B107] Ude S. Arnold D. L. Moon C. D. Timms-Wilson T. Spiers A. J. (2006). Biofilm formation and cellulose expression among diverse environmental *Pseudomonas* isolates. *Environ. Microbiol.* 8 1997–2011. 10.1111/j.1462-2920.2006.01080.x 17014498

[B108] Uhlich G. A. Cooke P. H. Solomon E. B. (2006). Analyses of the red-dry-rough phenotype of an *Escherichia coli* O157:H7 strain and its role in biofilm formation and resistance to antimicrobial agents. *Appl. Environ. Microbiol.* 72 2564–2572. 10.1128/AEM.72.4.2564-2572.2006 16597958 PMC1449024

[B109] van Hullebusch E. D. Zandvoor M. H. Lens P. N. L. (2003). Metal immobilization by biofilms: mechanisms and analytical tools. *Rev. Environ. Sci. Biotechnol.* 2 9–33. 10.1023/B:RESB.0000022995.48330.55

[B110] von Canstein H. Kelly S. Li Y. Wagner-Döbler I. (2002). Species diversity improves the efficiency of mercury-reducing biofilms under changing environmental conditions. *Appl. Environ. Microbiol.* 68 2829–2837. 10.1128/AEM.68.6.2829-2837.2002 12039739 PMC123942

[B111] Wagner-Döbler I. Lünsdort H. Lübbenhüsen T. von Canstein H. F. Li Y. (2000). Structure and species composition of mercury-reducing biofilms. *Appl. Environ. Microbiol.* 66 4559–4563. 10.1128/AEM.66.10.4559-4563.2000 11010917 PMC92343

[B112] Wang H. Yan Y. Rong D. Wang J. Wang H. Liu Z. (2016). Increased biofilm formation ability in *Klebsiella pneumoniae* after short-term exposure to a simulated microgravity environment. *Microbiologyopen* 5 793–801. 10.1002/mbo3.370 27185296 PMC5061716

[B113] Weiss-Muszkat M. Shakh D. Zhou Y. Pinto R. Belausov E. Chapman M. R. (2010). Biofilm by and multicellular behavior of *Escherichia coli* O55:H7, an atypical enteropathogenic strain. *Appl. Environ. Microbiol.* 7 1545–1554. 10.1128/AEM.01395-09 20080991 PMC2832381

[B114] Whitchurch C. B. Tolker-Nielsen T. Ragas P. C. Mattick J. S. (2002). Extracellular DNA required for bacterial biofilm formation. *Science* 295:1487. 10.1126/science.295.5559.1487 11859186

[B115] White A. Gibson D. L. Collinson S. K. Banser P. A. Kay W. W. (2003). Extracellular polysaccharides associated with thin aggregative fimbriae of *Salmonella enterica* serovar Enteritidis. *J. Bacteriol.* 185 5398–5407. 10.1128/JB.185.18.5398-5407.2003 12949092 PMC193744

[B116] Wingender J. Strathmann M. Rode A. Leis A. Flemming H.-C. (2001). Isolation and biochemical characterization of extracellular polymeric substances from *Pseudomonas aeruginosa*. *Methods Enzymol.* 336 302–314. 10.1016/S0076-6879(01)36597-7 11398408

[B117] Yamaga F. Washio K. Morikawa M. (2010). Sustainable biodegradation of phenol by *Acinetobacter calcoaceticus* P23 isolated from the rhizosphere of duckweed *Lemna aoukikusa*. *Environ. Sci. Technol.* 44 6470–6474. 10.1021/es1007017 20704249

[B118] Yap M.-N. Yang C.-H. Barak J. D. Jahn C. E. Charkowski A. O. (2005). The *Erwinia chrysanthemi* type III secretion system is required for multicellular behavior. *J. Bacteriol.* 187 639–648. 10.1128/jb.187.2.639-648.2005 15629935 PMC543537

[B119] Zabłocka-Godlewska E. Przystaś W. Grabińska-Sota E. (2012). Decolourization of diazo evans blue by two strains of *Pseudomonas fluorescens* isolated from different wastewater treatment plants. *Water Air Soil Pollut.* 223 5259–5266. 10.1007/s11270-012-1276-4 23002313 PMC3443478

[B120] Zhi S. Banting G. Li Q. Edge T. A. Topp E. Sokurenko M. (2016). Evidence of naturalized stress-tolerant strains of *Escherichia coli* in municipal wastewater treatment plants. *Appl. Environ. Microbiol.* 82 5505–5518. 10.1128/AEM.00143-16 27371583 PMC5007776

[B121] Zogaj X. Bokranz W. Nimtz M. Römling U. (2003). Production of cellulose and curli fimbriae by members of the family *Enterobacteriaceae* isolated from the human gastrointestinal tract. *Infect. Immun.* 71 4151–4158. 10.1128/IAI.71.7.4151-4158.2003 12819107 PMC162016

[B122] Zogaj X. Nimtz M. Rohde M. Bokranz W. Römling U. (2001). The multicellular morphotypes of *Salmonella typhimurium* and *Escherichia coli* produce cellulose as the second compound of the extracellular matrix. *Mol. Microbiol.* 39 1452–1463. 10.1046/j.1365-2958.2001.02337.x 11260463

[B123] Zou L. Zeng Q. Lin H. Gyaneshwar P. Chen G. Yang C.-H. (2012). SlyA regulates type III secretion system (T3SS) genes in parallel with the T3SS master regulator HrpL in *Dickeya dadantii* 3937. *Appl. Environ. Microbiol.* 78 2888–2895. 10.1128/AEM.07021-11 22267675 PMC3318817

