# Supplementary material for: Metal-Adapted Bacteria Isolated From Wastewaters Produce Biofilms by Expressing Proteinaceous Curli Fimbriae and Cellulose Nanofibers
Source: Front Microbiol. 2018 Jun 25;9:1334. doi: 10.3389/fmicb.2018.01334 (PMC6026672; doi:10.3389/fmicb.2018.01334)
Supplement: Supplementary file 1 [file Table_1.DOCX]

**Table 1** Physico-chemical properties of the wastewater samples

| Characteristics | Types of wastewater | | | | |
| --- | --- | --- | --- | --- | --- |
|  | Dyeing industry | Composite (Household plus mixture of industry) | Garments industry | Washing plant industry | Tannery industry |
| Color | Purple | Turbid | Black | Bluish | Brownish |
| Odor | Strongly pungent | Less pungent | Strongly Pungent | Strongly Pungent | Strongly Pungent |
| Temperature (°C) | 24.9 | 24.1 | 25.8 | 27.6 | 24.3 |
| pH | 7.13 | 7.29 | 7.50 | 8.03 | 7.31 |
| Total suspended solid (ppm) | 458 | 393 | 905 | 752 | 1526 |
| Salinity (ppm) | 500 | 400 | 800 | 800 | 1600 |
| Dissolve oxygen (mg/L) | 2.51 | 0.18 | 0.30 | 0.18 | 3.48 |
| Conductivity ((µS/cm) | 910 | 817 | 1670 | 1648 | 1350 |
| Heavy metals (mg/L) |  |  |  |  |  |
| Cu | 4.2 ± 0.02 | 0.9 ± 0.02 | 6.9 ± 0.8 | 3.7 ± 0.4 | 4.9 ±0.7 |
| Zn | 2.6 ± 0.03 | 1.1 ± 0.03 | 5.2 ± 0.2 | 1.3 ± 0.3 | 2.3 ± 0.6 |
| Pb | 0.3 ± 0.001 | 0.09 ± 0.001 | 0.5 ± 0.01 | 0.05 ± 0.01 | 0.2 ± 0.001 |
| Cr | 0.9 ± 0.02 | 0.02 ± 0.001 | 0.7 ± 0.02 | 0.03 ± 0.01 | 9.8 ± 0.8 |
| Ni | 0.6 ± 0.002 | 0.02 ± 0.001 | 0.7 ± 0.01 | 0.9 ± 0.2 | 1.3 ± 0.9 |
