# Supplementary figures and images for: Metal-Adapted Bacteria Isolated From Wastewaters Produce Biofilms by Expressing Proteinaceous Curli Fimbriae and Cellulose Nanofibers
Source: Front Microbiol. 2018 Jun 25;9:1334. doi: 10.3389/fmicb.2018.01334 (PMC6026672; doi:10.3389/fmicb.2018.01334)

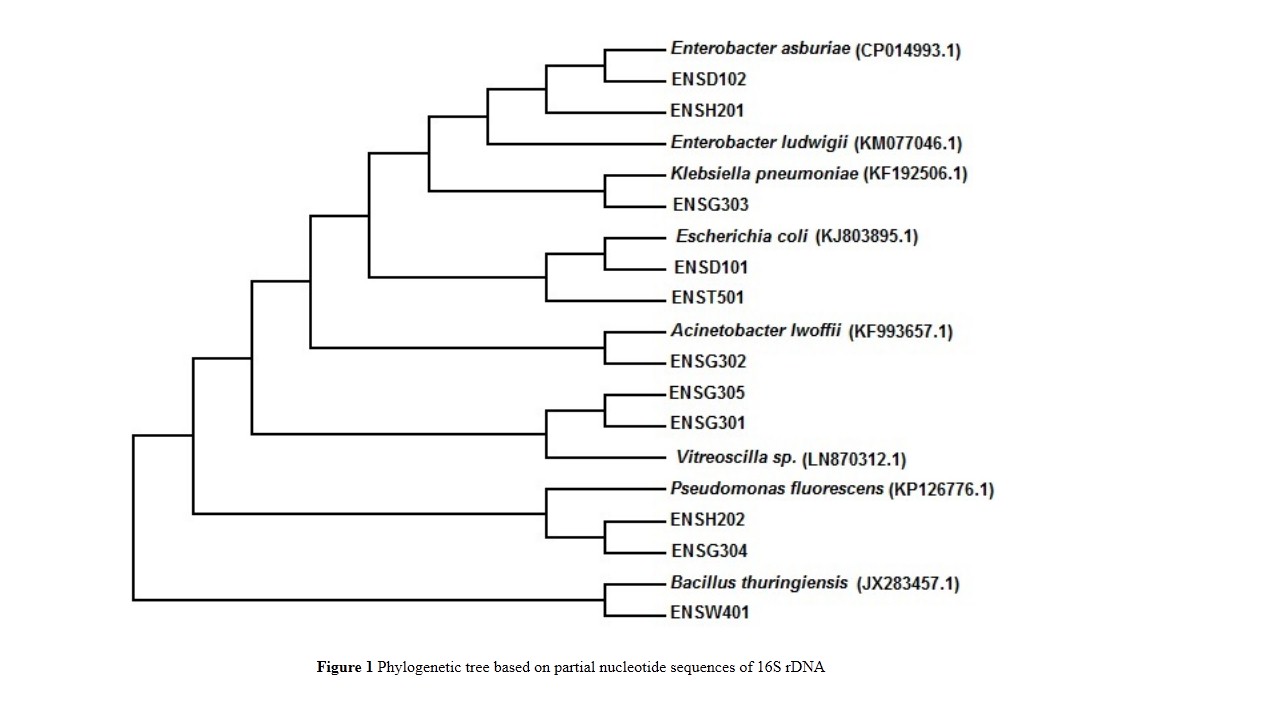

Supplement: Supplementary file 2 [file Image_1.JPEG]
